# Inflammasome activation: from molecular mechanisms to autoinflammation

**DOI:** 10.1002/cti2.1404

**Published:** 2022-07-07

**Authors:** Samuel Lara‐Reyna, Emily A Caseley, Joanne Topping, François Rodrigues, Jorge Jimenez Macias, Sean E Lawler, Michael F McDermott

**Affiliations:** ^1^ Institute of Microbiology and Infection University of Birmingham Birmingham UK; ^2^ School of Biomedical Sciences, Faculty of Biological Sciences University of Leeds Leeds UK; ^3^ Leeds Institute of Rheumatic and Musculoskeletal Medicine, St James's University Hospital University of Leeds Leeds UK; ^4^ AP–HP, Hôpital Tenon, Sorbonne Université, Service de Médecine interne Centre de Référence des Maladies Auto‐inflammatoires et des Amyloses d'origine inflammatoire (CEREMAIA) Paris France; ^5^ Harvey Cushing Neuro‐Oncology Laboratories, Department of Neurosurgery, Brigham and Women's Hospital Harvard Medical School Boston Massachusetts USA; ^6^ Brown Cancer Centre, Department of Pathology and Laboratory Medicine Brown University Providence Rhode Island USA

**Keywords:** autoinflammatory disorders, inflammasome, inflammation, NLRC4, NLRP3, Pyrin

## Abstract

Inflammasomes are assembled by innate immune sensors that cells employ to detect a range of danger signals and respond with pro‐inflammatory signalling. Inflammasomes activate inflammatory caspases, which trigger a cascade of molecular events with the potential to compromise cellular integrity and release the IL‐1β and IL‐18 pro‐inflammatory cytokines. Several molecular mechanisms, working in concert, ensure that inflammasome activation is tightly regulated; these include NLRP3 post‐translational modifications, ubiquitination and phosphorylation, as well as single‐domain proteins that competitively bind to key inflammasome components, such as the CARD‐only proteins (COPs) and PYD‐only proteins (POPs). These diverse regulatory systems ensure that a suitable level of inflammation is initiated to counteract any cellular insult, while simultaneously preserving tissue architecture. When inflammasomes are aberrantly activated can drive excessive production of pro‐inflammatory cytokines and cell death, leading to tissue damage. In several autoinflammatory conditions, inflammasomes are aberrantly activated with subsequent development of clinical features that reflect the degree of underlying tissue and organ damage. Several of the resulting disease complications may be successfully controlled by anti‐inflammatory drugs and/or specific cytokine inhibitors, in addition to more recently developed small‐molecule inhibitors. In this review, we will explore the molecular processes underlying the activation of several inflammasomes and highlight their role during health and disease. We also describe the detrimental effects of these inflammasome complexes, in some pathological conditions, and review current therapeutic approaches as well as future prospective treatments.

## Graphical abstract

This review explores the molecular processes underlying the activation of several inflammasomes and highlights their role during health and disease. We describe the harmful effects of these molecular complexes, in some autoinflammatory disorders, and review current therapeutic approaches as well as future prospective treatments.

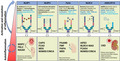

## Introduction

Innate immune cells constitute the first line of defence against invading pathogens, conferring host protection through several multidimensional non‐specific molecular strategies. Activation of these pathways normally occurs via detection of danger‐associated molecular patterns (DAMPs) and pathogen‐associated molecular patterns (PAMPs), such as lipopolysaccharide (LPS), which is an integral component of Gram‐negative bacteria outer membranes. LPS is recognised by several different pattern‐recognition receptors (PRRs),^1^ which induce a specific response to counteract each individual situation and the particular invading organism encountered. For instance, phagocytes can detect opsonised molecules, via their opsonic receptors, and these cells will eventually phagocytose the targeted molecule. In situations where the insult overcomes the initial innate defence response, a more complex, robust and damaging mechanism may be induced by activating the inflammasome. The inflammasome was first described, in 2002, by Martinon et al. as a ‘high molecular weight complex containing NALP1, Pycard, and proinflammatory caspases’^2^; as then several more studies have elucidated several distinct inflammasomes that can be assembled through activation of other cytosolic PRRs.^3^, ^4^ In general, inflammasomes are activated by two consecutive signals; the first signal arises from Toll‐like receptors (TLRs) that are activated by a PAMP such as LPS, which triggers the production of inactive forms of two pro‐inflammatory cytokines, interleukin‐1β (IL‐1β) and interleukin‐18 (IL‐18), known as pro‐IL‐1β and pro‐IL‐18.^2^ Cytokines such as TNF or even IL‐1 itself can also provide this first signal.^5^ The second signal can arise from multiple pathways (e.g. K^+^ efflux via P2X7 receptor activation, endoplasmic reticulum stress) and results in the activation of proinflammatory caspases, which in turn proteolytically activateIL‐1β and IL‐18 for secretion.^6^ Although caspase‐1 activation is essential to the inflammasome‐mediated cleavage of both pro‐IL‐1β and pro‐IL‐18, a number of different proteases, apart from caspase‐1, may also cleave the pro‐forms of IL‐1β and IL‐18 at different sites, thereby giving rise to peptide/protein fragments with non‐inflammasome‐related biological activity.^6^, ^7^ Nevertheless, not all inflammasomes need the priming signal to become fully activated and they can induce the secretion of inflammatory cytokines through other mechanism.

**Figure 1:**
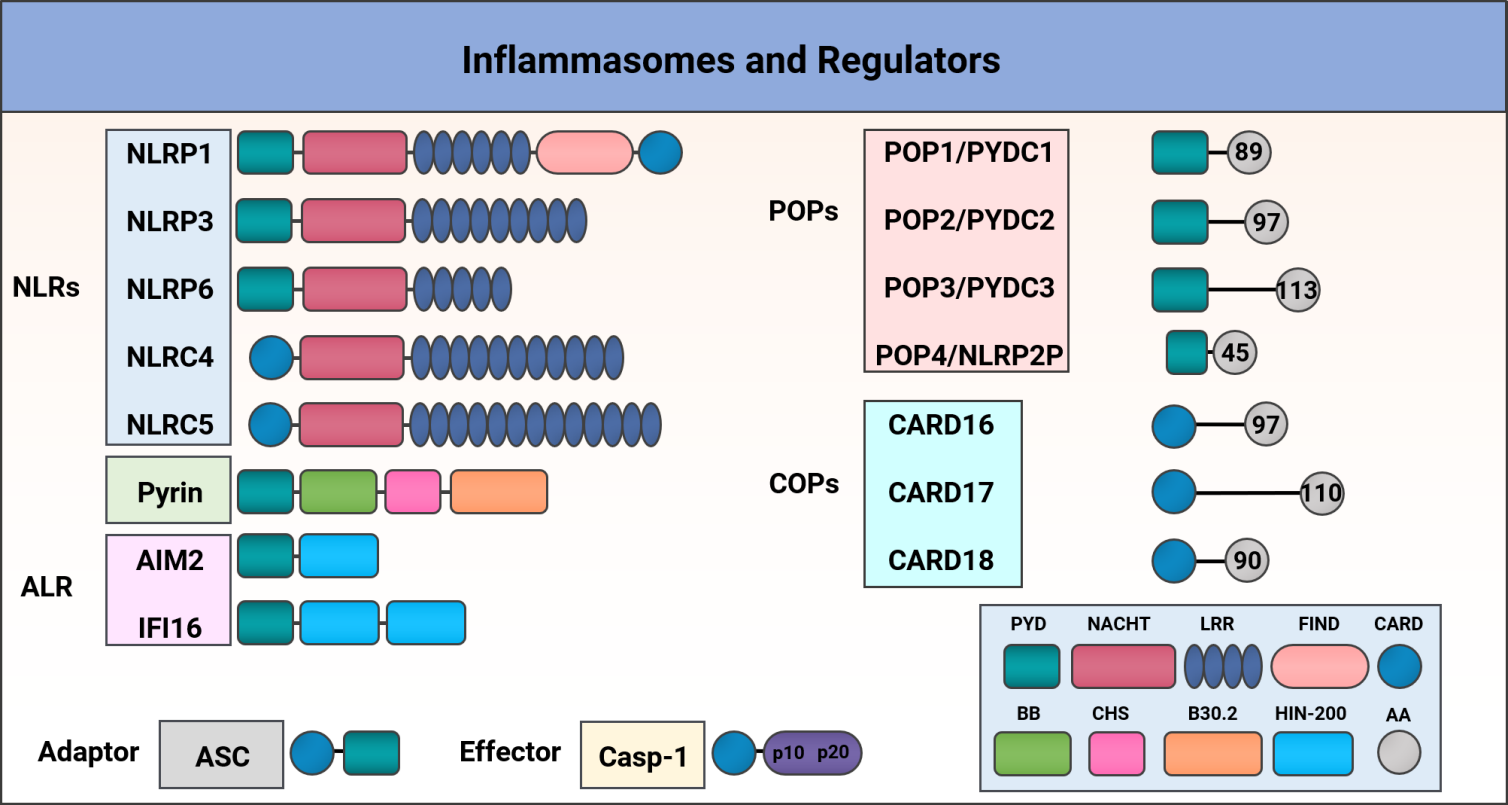
Domain structure of inflammasomes and regulators. Different types of inflammasomes are shown; Nucleotide‐binding and oligomerisation domain NOD‐like receptors (NLRs): NLRP1, NLRP3, NLRP6, NLRC4 and NLRC5 all contain a central NACHT domain and a and C‐terminal leucine‐rich repeats (LRRs) domain; NLRP1, NLRP3, NLRP6 encompass an N‐terminal Pyrin domain (PYD); NLRP1, NLRC4 and NLRC5 contain a caspase recruitment domain (CARD); only the human NLRP1 contains a function‐to‐find domain (FIIND). Pyrin inflammasome consists of a PYD, a B‐box (BB), a coiled‐coil (CC), and a B30.2 domain. The AIM2‐Like Receptors (ALR) AIM2 and IFI16 are members of PYHIN family, containing an N‐terminal PYD and C‐terminal HIN‐200 domain. PYD‐only proteins (POPs) and CARD‐only proteins (COPs) are composed of PYD or CARD domains, accordingly, with different amino acid (AA) composition. The adaptor protein ASC links inflammasome sensors and caspase‐1 via the PYD and CARD domains.

Several inflammasomes with a range of different PRRs, functions and regulators are now described.^3^, ^4^ Generally speaking, ‘canonical’ inflammasomes can be classified according to the three different categories of PRRs that nucleate these protein complexes; these are NOD‐like receptors (NLRs), pyrin and AIM2‐like receptors (ALRs) (Figure 1). Inflammasome sensor activation is usually followed by recruitment of the adaptor molecule apoptosis‐associated speck‐like protein containing a CARD (ASC).^8^ Finally, the effector molecule, caspase‐1, is recruited to the complex by ASC to form the large multimeric inflammasome complex.^9^ Inflammasomes usually employ caspase‐1 as their effector protein; however, ‘non‐canonical inflammasomes’ are also described, which activate other caspases, such as caspases‐11/4/5.^10^ These specialised ‘non‐canonical’ inflammasomes can detect intracellular LPS inducing inflammatory responses and pyroptosis.^11^

While detection of PAMPs and DAMPs by specific inflammasome sensor proteins is usually followed by a pro‐inflammatory response, two other single‐domain protein molecular families, referred to as CARD‐only proteins (COPs) and pyrin‐only proteins (POPs), have emerged as negative regulators of inflammasomes^12^ (Figure 1). These short structures are important inflammasome regulators and play a key role in fine‐tuning the inflammatory response.

Inflammasomes are vital innate immune defence complexes, responsible for monitoring pathogenic invasions and counteracting molecular insults. Perturbations of their signalling mechanisms may lead to immunological conditions^13^, ^14^ and genetic disorders.^13^, ^15^ Autoinflammatory diseases, such as tumor necrosis factor receptor‐associated periodic syndrome (TRAPS) and familial mediterranean fever (FMF), have all been directly linked with excessive production of IL‐1β and IL‐18,^14^ because of heightened inflammasome responses. Therefore, a selection of these conditions will also be discussed, from the molecular level to the clinical picture, including current and potential future treatments.

## Inflammasomes signalling pathways

**Figure 2:**
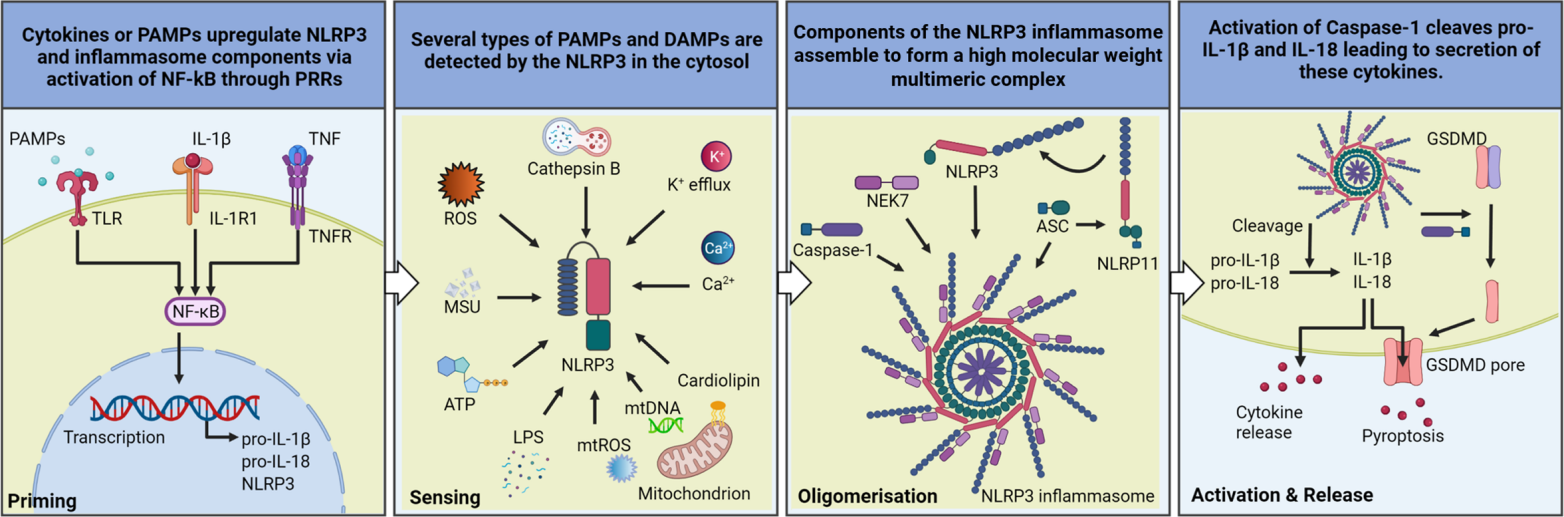
NLRP3 inflammasome activation. Danger‐associated molecular patterns (DAMPs) or pathogen‐associated molecular patterns (PAMPs) are able to induce the priming step inducing the transcriptional upregulation of inflammasome components. DAMPs or PAMPs are subsequently sensed inducing oligomerisation of the inflammasome sensor, NLRP3. Caspase‐1, NEK7, ASC and the NLRP3 all together form the NLRP3 inflammasome. The NLRP11bound to ASC is required for NLRP3 oligomerisation and ASC polymerisation. The inflammasome is activated and active caspase‐1 cleaves pro‐IL‐1β and pro‐IL‐18 to their mature forms IL‐1β and IL‐18 which get secreted through gasdermin D pores (GSDMD); alternatively, IL‐1β and IL‐18 can be secreted via different mechanism avoiding GSDMD.

While inflammasome activation may vary in character, depending on the inflammasome in question, the activation process essentially involves four key steps overall – priming, sensing, oligomerisation and activation/release of inflammatory cytokines^16^ (Figure 2). In the priming step, PAMPs and/or DAMPs induce transcriptional upregulation of specific components of the inflammasome, such as caspases, pro‐IL‐1β and pro‐IL‐18.^17^ This first priming step is a ‘get‐ready’ signal for the cell to be alert in case a specific inflammasome requires activation. If the cell is subject to a sensing signal, then inflammasomes sense intracellular insults and this is followed by a third step involving inflammasome assembly. Finally, the whole process culminates in release of pro‐inflammatory cytokines through membrane pores formed by the pore‐forming protein, gasdermin D (GSDMD), while simultaneously inducing pyroptosis. While pyroptosis is a consequence of inflammasome activation, other types of cell death, such as apoptosis, necroptosis and PANoptosis, have also been associated with inflammasome activation^16^, ^17^, ^18^ (Figure 2). As an example, the NLRP3 inflammasome activation is shown, nevertheless other inflammasomes may signal exactly like the NLRP3 and these will be described in their sections. For example, LPS can function as one of the first priming steps in certain inflammasomes, by inducing the transcription of inflammasome components, such as NLRP3, caspase‐1, pro‐IL‐1β and pro‐IL‐18. On subsequent detection of specific DAMPs or PAMPs, such as ATP, urate crystals, bacterial‐, viral‐ or fungal‐ derived components, oligomerisation of the NLRP3 inflammasome occurs with binding of NIMA‐related kinase 7 (NEK7) and subsequent recruiting of ASC and caspase‐1 to facilitate dimerisation, cleavage and activation of caspase‐1.^16^ Active caspase‐1 induces the cleavage of inflammasome‐specific cytokines, such as IL‐1β and IL‐18, and directs their secretion via pores in the plasma membrane generated by GSDMD, inducing pyroptosis^19^, ^20^ (Figure 2).

While the activation mechanism of the inflammasomes aforementioned is well accepted within the majority of them, several other mechanisms exist that can induce inflammasome assembly and caspase activation. For instance, NLRP1 and CARD8 can undergo post‐translational autoproteolysis inducing inflammasome activation and release of IL‐1β and IL‐18 ASC‐independently.^21^, ^22^ Similarly, NLRC4 can directly interact with caspase‐1 via its CARD domain, causing protease activation and release of proinflammatory cytokines.^23^ Furthermore, these ASC‐independent events induce caspase‐1 activation, and these processes are enhanced by a priming step which induces ASC and other caspase substrates.^23^, ^24^

## NLRP1

The first inflammasome to be described contained NOD‐, LRR‐ and pyrin domain‐containing protein 1 (NLRP1).^2^ This inflammasome contains a unique function‐to‐find domain (FIIND), located after its LRR domain (Figure 1). FIIND is a highly conserved protein region present in the NLRP1 and CARD8, and its function is still to be fully elucidated^2^, ^25^; however, a study has shown that FIIND is a type of ZU5‐ and UPA‐like domain contained in autoproteolytic proteins, which undergo autocleavage.^26^ The FIIND domain of NLRP1 interacts with dipeptidyl peptidases 8 and 9 (DPP8 and DPP9), suppressing NLRP1 activation.^27^ DPP8 and DPP9 are cytosolic prolyl peptidases, with the ability to cleave proline bonds from the N‐terminus of substrates, important in the regulation of immune cell functions. The NLRP1 and DDP9 proteins form a complex containing the inhibited NLRP1 molecule and an active UPA‐CARD NLRP1 fragment^28^; the ZU5 domain is essential for both, formation of the complex and autoinhibition of NLRP1.^28^ NLRP1 haplotype single‐nucleotide polymorphisms (SNPs) (M1184V) were associated with reduction of inflammasome activation, via increased binding of DPP9 to the mutated FIIND domain, and this was associated with increased asthma severity.^29^ Another unique characteristic of the NLRP1 inflammasome is the location of its CARD domain in its C‐terminal region, where it activates caspase‐1. Although the NLRP1 inflammasome can be found in most cell types and tissues, it appears to have a vital role in epithelial barriers, particularly in the integumentary and respiratory systems.^30^, ^31^, ^32^

The recently described CARD8 inflammasome, containing a short N‐terminal region, a FIIND, and a CARD domain, can also trigger pyroptosis in CD4^+^ and CD8^+^ T cells regulated by DPP9.^33^ Furthermore, CARD8 was shown to detect HIV‐1 protease activity and induce CARD8‐depedent pyroptosis in infected T cells and macrophages.^34^ The NLRP1 and CARD8 inflammasomes are repressed by DPP8/9 via different mechanisms.^35^ A recent study shows that a small molecule named CQ31 can specifically activate CARD8 via inhibition of M24B aminopeptidases prolidase (PEPD) and Xaa‐Pro aminopeptidase 1 (XPNPEP1).^35^ This inhibition leads to accumulation of proline‐containing peptides that repress DPP8/9 inducing CARD8 activation.

NLRP1 detects multiple viral protease activities^36^, ^37^ in addition to detecting double‐stranded (ds) RNA, via its LRR domain, and effectively sensing virus‐associated nucleic acids.^38^ Both human and mouse NLRP1 are cleaved in a similar manner, leading to degradation of the N‐terminal fragment, with liberation of the C‐terminal fragment containing the CARD domain, a potent activator of caspase‐1.^36^, ^39^ In this way, NLRP1 is seen to be a potent inducer of IL‐18 and IL‐1β, and fundamental to host protection against certain viruses, mainly in epithelial barriers.

Germline mutations in NLRP1 have been reported to be responsible for two overlying monogenic skin disorders, multiple self‐healing palmoplantar carcinoma (MSPC) and familial keratosis lichenoides chronica (FKLC).^30^ In that particular study, the reported NLRP1 mutations were gain‐of‐function mutations, predisposing epithelial cells to NLRP1 inflammasome activation, and augmenting the levels of IL‐1β, which exerted paracrine signalling effects leading to multiple epithelium‐associated clinical symptoms.^30^ MSPC and FKLC both demonstrate classical features of autoinflammation, with spontaneous activation of inflammatory pathways which, in theory, could be resolved by IL‐1β inhibition. Moreover, a total of 23 NLRP1 SNPs have been associated with the vitiligo phenotype with associated autoimmune and/or autoinflammatory features.^31^ Intriguingly, only gain‐of‐function mutations have been reported, so far, in relation to the NLRP1 inflammasome, with another study presenting two cases of juvenile‐onset recurrent respiratory papillomatosis, with high levels of IL‐18, but not of IL‐1β, in patients' serum.^32^ Discrepancies in IL‐18 and IL‐1β cytokine profiles are observed in other autoinflammatory disorders, with potential divergent roles regarding the site of inflammation.^40^, ^41^ These IL‐18 and IL‐1β disparities can be clinically relevant for the selective treatment of certain inflammatory symptoms observed in some autoinflammatory conditions via small‐molecule drugs. Although NLRP1 was the first inflammasome to be described, several questions remain regarding its function and role in other immune‐related disorders.

## NLRP3

The NOD‐, LRR‐ and pyrin domain‐containing protein 3 (NLRP3) inflammasome plays a critical role in host defence against microbial infections and a multitude of other harmful stimuli in response to endogenous stress. NLRP3 is tightly regulated by post‐transcriptional and post‐translational modifications and a variety of endogenous modulators.^42^, ^43^ Under aberrant conditions, the NLRP3 inflammasome is associated with many autoinflammatory and metabolic disorders, including cryopyrin‐associated periodic syndromes (CAPS), myelodysplastic syndromes (MDS), obesity, type‐2 diabetes and Alzheimer's disease.^44^

It is generally thought that effective NLRP3 inflammasome function requires both ‘priming’ and activation, as described above (Figure 2); however, a recent study found that a priming step may be dispensable in human monocytes, but essential in monocyte‐derived macrophages.^45^, ^46^ These findings are worth considering while developing specific inflammasome inhibitors, for the purpose of targeting specific steps in inflammasome activation. NLRP3 inflammasome activation involves TLR signalling to activate NF‐κB and induce pro‐IL‐1β and NLRP3 expression, and subsequent activation of the NLRP3 inflammasome by plasma membrane disruption (for bacterial toxins) or by internalisation of particulate activators, such as alum or monosodium urate crystals by phagocytosis.^43^ NLRP3 undergoes conformational changes upon activation, mediated by RACK1 and binding to NEK7.^47^ This enables oligomerisation, providing a platform for ASC recruitment, via PYD‐PYD interactions.^47^ ASC subsequently polymerises to form large cytoplasmic filaments called ASC specks, and recruits caspase‐1 (through its CARD domain), which undergoes dimerisation, self‐cleavage at the interdomain linker, and subsequent increase in caspase‐1 catalytic activity.^48^ This domino effect results in cleavage, maturation and secretion of IL‐1β, IL‐18, as well as liberation of GSDMD's N‐terminal domain, to initiate pyroptotic cell death (Figure 2).

Despite extensive NLRP3 inflammasome characterisation, the precise mechanisms of activation and regulation remain unknown: nevertheless, potential therapeutic targets are constantly emerging.^49^, ^50^ The cGAS‐STING pathway was recently shown to recruit NLRP3 at the endoplasmic reticulum upon HSV‐1 infection, with subsequent NF‐κB and NLRP3 activation.^51^ Specific blockade of the NLRP3 inflammasome, but not the AIM2 or NLRC4 inflammasomes, by itaconate and its derivative 4‐OI, leads to modification of cysteine 548 in the NLRP3 molecule, thereby interfering with NLRP3‐NEK7 interactions.^52^ Itaconate is a potent anti‐inflammatory and anti‐microbial metabolite, produced by immune‐responsive gene 1 (IRG1) enzyme,^52^, ^53^ and itaconate‐depleted *lrg1* ^−/−^ macrophages show increased NLRP3 activation.^52^

There is increasing evidence of the NLRP3 inflammasome's role in sensing bacterial toxins and virulence factors and activating host Rho GTPases. p21‐activated kinases 1 and 2 (Pak1/2) and the Pak1‐mediated phosphorylation of Thr‐659 of NLRP3 were shown to essential for the NLRP3‐Nek7 interaction in mice.^54^ In a different study carried out in mouse and human macrophages, recruitment of NEK7 to NLRP3 was controlled by phosphorylation of Serine‐803 (S803) in the LRR domain of NLRP3 (S806 in humans).^55^ These findings suggest an important regulatory mechanism targetable by small molecules, to disrupt NLRP3 inflammasome assembly.

The majority of stimuli that activate the NLRP3 inflammasome do not directly bind to NLRP3^56^, ^57^ and mitochondria have been proposed as the missing link underlying this mechanism. Mitochondria can be damaged by danger signals, such as K^+^ efflux or changes in intracellular Ca^2+^, resulting in mitochondrial DNA (mtDNA) release into the cytosol, triggering reactive oxygen species (ROS) and oxidisation of the mtDNA, which may become an NLRP3 ligand.^58^, ^59^In support of this model of ROS‐modified mtDNA as an NLRP3 activator, macrophages lacking mtDNA show reduced IL‐1β production^58^; however, questions remain about the high concentration of ROS inhibitors used, leading to potential artefacts or inhibition of NLRP3 priming.^43^, ^60^ Nevertheless, LPS triggers MyD88 and TRIF signalling, and activates IRF1‐dependent transcription of CMPK2 (a mitochondrial nucleotide kinase) to enable mtDNA synthesis.^61^, ^62^ This newly synthesised mtDNA is particularly susceptible to oxidisation, and may release fragments of ox‐mtDNA into the cytosol to co‐localise with components of the NLRP3 inflammasome in macrophages.^61^ This co‐localisation of NLRP3 induced ASC‐specks and ox‐mtDNA, however, is not observed with AIM2‐induced ASC specks.^61^ Associations between circulating mtDNA and NLRP3 are also emerging, with potential as biomarkers of NLRP3 activation and increased levels of cell‐free circulating mtDNA have been linked to a poor prognosis in patients with COVID‐19.^63^ Indeed, coronaviruses have been shown to activate the NLRP3 inflammasome, via either indirect interaction with NLRP3 (SARS‐CoV E viroporin protein, viroporin 3a) or direct activation with NLRP3 (SARS‐CoV‐2 N protein), resulting in hyperinflammation and increased COVID‐19 severity.^64^, ^65^, ^66^ Finally, guanylate‐binding proteins (GBPs), members of the GTPase family, are associated in the regulation of several inflammasomes.^67^ Increased levels GBPs have been reported in certain inflammatory and autoimmune disorders with important implications for the NLRP3.^68^ In the context of NLRP3, human GBP5 binds the pyrin domain of the NLRP3 increasing complex formation.^69^ Moreover, Gbp5^−/−^ bone marrow‐derived macrophages show impaired activation of the NLRP3 inflammasome, but not NLRC4, with low levels of IL‐1β and IL‐18.^69^

## NLRP 6 and 7

The NLRP6 inflammasome has been implicated in a number of different physiological processes, including host defence against microbial infections,^70^, ^71^, ^72^, ^73^ maintenance of epithelial integrity^73^, ^74^ and regulation of neuroinflammation.^75^, ^76^ The initial characterisation of NLRP6 identified it as having a unique property among the NLRs in that it activates both caspase‐1 and NF‐κB,^77^ although, interestingly, it has also been shown to negatively regulate NF‐κB downstream of TLR signalling in a mouse model.^72^

Evidence strongly suggests that NLRP6 assembles an inflammasome. NLRP6 co‐localises with ASC, both under steady‐state conditions and following bacterial infection *in vitro*.^70^, ^77^, ^78^ *In vivo* studies showed reduced serum IL‐18 levels in Nlrp6^−/−^ mice, both under steady‐state conditions and following dextran sulphate sodium (DSS)‐induced colitis than in WT mice,^79^, ^80^ as well as reduced IL‐1β levels in bronchoalveolar lavage fluid from Nlrp6^−/−^ mice compared with WT following MRSA infection.^70^ However, in these same studies, IL‐1β expression was increased in the colon of Nlrp6^−/−^ mice^79^ and both IL‐1β levels and caspase‐1 activity were comparable between Nlrp6^−/−^ and WT mice following infection with various bacteria,^72^ making it unclear whether NLRP6 inflammasome assembly occurs during systemic bacterial infection.

More recently, the NLRP6 inflammasome was shown to be activated by Gram‐positive bacteria, including *Staphylococcus aureus* (MRSA) and *Listeria monocytogenes*, leading to IL‐1β and IL‐18 maturation and pyroptosis.^70^, ^71^, ^81^ Lipoteichoic acid (LTA) has been implicated in the initial ‘priming’ signalling event; it has been found to upregulate NLRP6 and caspase‐11 expression via type 1 interferon signalling,^71^ as well as activating the NLRP6 inflammasome through the ASC‐caspase‐11‐caspase‐1 signalling pathway.^71^

The NLRP7 inflammasome is also incompletely characterised, with evidence suggesting that to recognises cell wall components and microbial acylated lipopeptides, and promotes ASC‐dependent activation of caspase‐1, with IL‐1β and IL‐18 maturation to restrict intracellular bacterial replication.^82^ Activation of this proposed inflammasome following *Staphylococcus aureus* infection has been suggested to require ATP binding at the NLRP7 Walker A motif,^83^ as well as being regulated by NLRP7 ubiquitination.^84^

Although further study defining the NLRP7 inflammasome is required, there is also substantial evidence to support NLRP7 as a negative regulator of inflammasome signalling. Reduced IL‐1β release has been observed in NLRP7 overexpression studies in HEK293 cells^77^, ^85^ and in peripheral blood mononuclear cells (PBMCs) from patients with disease‐associated loss‐of‐function *NLRP7* variants,^86^ whereas *NLRP7* mutants that resulted in truncated NLRP7 protein did not inhibit IL‐1β release.^87^, ^88^ Various mechanisms have been proposed for this regulation of IL‐1β, including direct interaction with and inhibition of inflammasome components, and alteration of pro‐IL‐1β transcription and subsequent trafficking and release of the mature forms,^89^ although further validation is required for this, as various reports have also shown a lack of effect of NLRP7 on inflammatory responses.^77^, ^82^, ^84^, ^90^ Taken together, these studies illustrate a multifaceted role for the NLRP7 inflammasome in the regulation of inflammatory responses. Taken together, these studies illustrate a multifaceted role for the NLRP7 in the regulation of inflammatory responses, wherein it may negatively regulate the inflammasome in the resting state but assemble an inflammasome following a stimulus such as bacterial infection.

## Pyrin

Similar to NLRP3, the pyrin inflammasome detects intracellular pathogens and is tailored to counteract bacterial toxins from different species.^91^ The human pyrin protein is composed of four main domains: an N‐terminal PYD domain which interacts with ASC with subsequent activation of caspase‐1: a B‐box domain 60 to 280 amino acids long; a central helical scaffold (CHS) connected to the B30.2 domain on its C‐terminus (Figure 1),^92^ and the B30.2 domain, where the majority of pathogenic mutations are found in humans, but this domain is absent in mice. The Pyrin inflammasome sensor detects inhibition of RhoA subfamily GTPases activity and is activated by various RhoA inhibiting toxins, including TcdA and TcdB from *Clostridioides difficile* (*C. difficile*) RhoA is a member of the GTPases superfamily that activates protein kinase N1 (PKN1) and PKN2, thereby facilitating binding of the inhibitory protein 14–3‐3 to pyrin by phosphorylating pyrin at Ser208 and Ser242, which results in the inactive form of pyrin.^91^, ^92^, ^93^ This inflammasome is triggered when bacterial toxins, such as *C. difficile* toxin A or B,^91^ inactivate RhoA, reducing PKN1 and PKN2 activity, dissociating 14–3‐3 from pyrin^93^ and fully activating pyrin, to culminate in caspase‐1 dimerisation and cleavage to caspase‐1 and production of mature forms of IL‐18 and IL‐1β.^94^ Aberrant actin depolymerisation can activate the pyrin inflammasome inducing autoinflammation driven by IL‐18, but not IL‐1β.^95^ Mice homozygous for a hypomorphic allele of Wdr1 manifested several cutaneous features which were similar to those presented by patients with autoinflammatory disorders. Finally, this unique pyrin inflammasome activation, driven by aberrant actin depolymerisation, was only observed in monocytes and not in macrophages or neutrophils.

**Figure 3:**
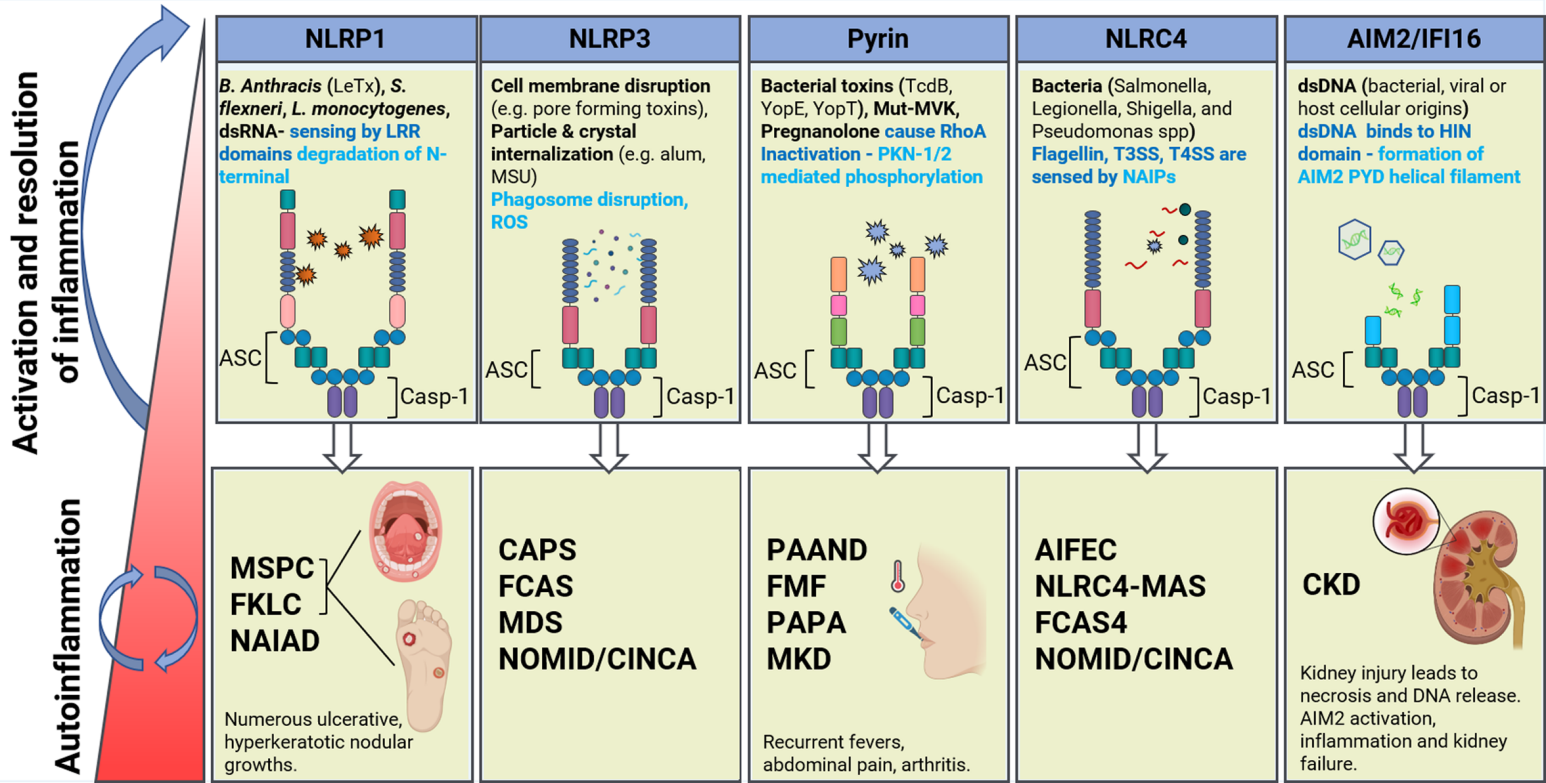
Inflammasome sensors, activators and related disorders. The NLRP1, NLRP3, pyrin, NLRC4 and AIM2/IFI16 with their current ligands and intracellular mediators involved in activation of these molecules. NLRP1 detects lethal toxin *Bacillus anthracis* (*B. anthracis*), *Shigella flexneri (S. flexneri)*, *Listeria monocytogenes (L. monocytogenes)*. NLRP3 is an overall sensor for several PAMPs and DAMPs (Figure 2) responding to intracellular damage induced by pathogenic or sterile insults. Pyrin inflammasome is activated by bacterial toxins that modify RhoA GTPases. The NLRC4 inflammasome detects bacterial proteins *via* NLR family‐apoptosis inhibitory proteins (NAIPs) and can assemble an inflammasome with or without ASC. Absent in melanoma 2 (AIM2) and IFNγinducible protein‐16 (IFI16) detect dsDNA via their HIN‐200 domains. When these sensors are chronically activated or not properly regulated, inflammatory‐related conditions are caused by these inflammasomes. MSPC, Multiple self‐healing palmoplantar carcinoma; FKLC, Familial keratosis lichenoides chronica; NAIAD, NLRP1‐associated autoinflammation with arthritis and dyskeratosis; MWS, Muckle–Wells syndrome; FCAS, familial cold autoinflammatory syndrome; MDS, myelodysplastic syndromes; neonatal‐onset multisystem inflammatory disease (NOMID)/chronic infantile neurological nutaneous and articular (CINCA); PAAND, pyrin associated autoinflammation with neutrophilic dermatosis; FMF, familial mediterranean fever; PAPA, pyogenic arthritis, pyoderma gangrenosum, and acne; MKD, mevalonate kinase deficiency; AIFEC, autoinflammation and infantile enterocolitis; NLRC4‐MAS, NLRC4 macrophage activation syndrome; CKD, chronic kidney disease.

Several autoinflammatory disorders have been linked, directly or indirectly, to the pyrin inflammasome^14^, ^96^ and will be discussed later (Figure 3). Mutations in the *MEFV* gene, which encodes pyrin in humans, are known to cause FMF.^97^ Receptor‐interacting serine/threonine‐protein kinase 3 (RIPK3) was recently shown to influence pyrin activation by exerting an inhibitory effect on the mammalian target of rapamycin (mTOR), leading to transcription of *MEFV* and pyrin inflammasome activation.^98^ Another study has demonstrated that while pyrin dephosphorylation was not sufficient to induce its activation in healthy individuals, the opposite was the case in FMF patients, with inflammasome activation, IL‐1β secretion and pyroptosis in monocytes.^99^ These molecular insights are of great importance in several autoinflammatory disorders and may form basis of new treatments for patients harbouring disease‐causing mutations. Activation of the pyrin inflammasome can also be induced by two microbiome‐derived bile acid metabolites, BAA485 and BAA473, revealing an unexplored role of pyrin in the lower gastrointestinal tract.^100^ Finally, while many *MEFV* mutations cause FMF, a recent study also showed that certain *MEFV* mutations, such as MEFV_p.M680I, confer protection against *Y. pestis* infection by inducing high levels of IL‐1β. Therefore, it is proposed that perhaps *Y. pestis* influenced evolutionary selection of some FMF‐associated mutations in certain human populations.^101^

## NAIP‐NLRC4

The NLR family CARD domain‐containing 4 (NLRC4) inflammasome is activated by bacterial flagellin and T3SS secretion system rod proteins and forms an integral component of the immune response against cytoplasmic Gram‐negative bacteria.^102^ In contrast to other inflammasomes, regulation by the NLRC4 inflammasome is dependent on its cooperative activity with NLR apoptosis inhibitory proteins (NAIPs), an NLR subfamily of proteins that act as cytosolic innate immune receptors for specific bacterial ligands. Whilst the mouse genome encodes NAIP1, NAIP2 and NAIP5/6,^103^, ^104^, ^105^, ^106^ there is only one human NAIP and this recognises the T3SS needle protein.^107^ Following detection of the relevant ligands, NAIPs bind to NLRC4 to induce a structural reorganisation from its autoinhibited state to its active configuration,^108^ which in turn recruits caspase‐1^109^; NLRC4 can bind to caspase‐1 directly via its CARD domain or indirectly via the adaptor molecule ASC that functions as an intermediary between NLRC4 and caspase‐1.^109^ Caspase‐1 activation leads to IL‐1β and IL‐18 release, with IL‐1β stimulating the recruitment of neutrophils to destroy extracellular bacteria. Caspase‐1 also initiates removal of intracellular bacteria via pyroptotic cell death, releasing the bacteria and exposing them to uptake and killing by ROS in neutrophils.^110^, ^111^

Pyroptotic bacterial clearance has led to further investigation of the role of NLRC4 in PANoptosis, an inflammatory programmed cell death mechanism controlled by a multimeric protein called the PANoptosome. This large complex comprises molecules from the programmed cell death pathways of pyroptosis, apoptosis and necroptosis,^112^ and NLRC4 has been implicated in caspase‐mediated cleavage of the DNA damage sensor poly (ADP‐ribose) polymerase 1 (PARP1), a hallmark of apoptosis.^113^ Furthermore, *Salmonella typhimurium* (*S. typhimurium*) infection induces both NLRC4 inflammasome activation and alongside other forms of cell death in macrophages.^114^ *S. typhimurium* clearance is also dependent on either caspase‐8 driven apoptotic, pyroptotic or necroptotic pathways, demonstrating cellular flexibility to protect against intracellular infections.^115^ As long as caspase‐1 or caspase‐8 are activated during *S. typhimurium* infection, macrophages can undergo a form of cell death to control *S. typhimurium* infection, which can be driven by the NLRC4 or other unknown factors.^115^

Four clinical phenotypes of NLRC4 inflammasome‐related diseases, because of gain‐of‐function *NLRC4* mutations, have been reported; autoinflammation and infantile enterocolitis (AIFEC), NLRC4 macrophage activation syndrome (NLRC4‐MAS), familial cold autoinflammatory syndrome (FCAS) and chronic infantile neurological cutaneous and articular (CINCA), also known as neonatal‐onset multisystem inflammatory disease (NOMID).^116^ However, NOMID/CINCA is often more associated with NLRP3 mutations. The severity of symptoms range from AIFEC and NLRC4‐MAS, which require frequent hospitalisations for life‐threatening episodes, to FCAS4 with milder phenotypes of urticarial rash and arthritis.^117^, ^118^ However, progress in this field is slow as there are relatively few known patients with pathogenic *NLRC4* mutations.^119^

## AIM2

Absent in Melanoma 2 (AIM2) is a cytosolic sensor that recognises double‐stranded DNA, binding through its C‐terminal domain HIN‐200 domain, and resulting in AIM2 inflammasome activation.^120^ Ongoing research shows that AIM2 is multifunctional, acting as a host defence against a range of pathogens, and also been implicated in a multitude of diseases.^120^, ^121^ Like the NLRP3 inflammasome, the AIM2 inflammasome pathway involves AIM2 activation and oligomerisation, ASC binding (through AIM2 and ASC PYDs), caspase‐1 dependent cleavage and activation of IL‐1β, IL‐18 and GSDMD, and subsequent pyroptosis.^121^ The assembly of AIM2‐ASC has also been implicated in caspase‐8 and downstream caspase‐9 activation causing caspase‐3 dependent, caspase‐1 independent, apoptosis.^122^ AIM2 inflammasome signalling has been shown to play a key role in neurodevelopment and CNS homeostasis, through its role in GSDMD regulation.^123^ AIM2 also has a role in early brain injury, following subarachnoid haemorrhage, as patients had significantly increased cerebral spinal fluid (CSF) levels of AIM2, and neuronal damage was reversed in AIM2 and caspase‐1 KO models.^124^ An additional role of AIM2 has emerged, linked to a regulatory role of pyrin and ZBP1, with the ability to induce PANoptosis through multi‐protein interactions of AIM2, pyrin, ZBP1, ASC, caspase‐1 and ‐8, RIPK‐3 and ‐1 and FADD through pyroptosis, apoptosis and necroptosis pathway interactions, following herpes simplex virus 1 (HSV‐1) and *Francisella novicida* (*F. novicida*) infection.^125^ Despite AIM2's major role in cytosolic DNA detection and inflammasome activation, this is dispensable in human myeloid cells; instead, the cGAS‐STING pathway is the main perpetrator of lysosomal cell death, by inducing NLRP3 activation, resulting from upstream K^+^ efflux and initiating antiviral immunity.^126^ Moreover, AIM2 senses radiation‐induced DNA damage, in intestinal epithelial and bone marrow cells, thereby activating the inflammasome with subsequent cellular death.^127^ This has therapeutic implications for AIM2 manipulation and control of inflammation. Recently, AIM2 was shown to play an important role in driving aldosterone‐induced renal injury via ER stress, with important implications in the treatment of chronic kidney disease (CKD).^128^

High‐mobility group box‐1 (HMGB1) has been implicated in AIM2‐ASC inflammasome activation.^129^ Circular RNA PPP1CC was recently shown to stimulate *Porphyromonas gingivalis* LPS‐induced pyroptosis in vascular smooth muscle cells, by AIM2 activation through the HMGB1/TLR9/AIM2 pathway, also promoting atherosclerosis.^130^ This study reported *in vitro* siRNA knockdown of circPPP1CC in HUVEC cells, resulting in pyroptosis decrease and reduced levels of HMGB1, TLR9, AIM2 and caspase‐1. HMGB1 has been implicated in LPS‐induced acute lung inflammation by activating the TLR2, TLR4 and RAGE/NF‐κB pathways in mice, with AIM2 inflammasome activation and M1 macrophage polarisation.^131^

GBP2 and GBP5 are important promoters of AIM2 inflammasome activation by inducing cytosolic bacteriolysis and DNA release during *F. novicida* infection.^132^ *F. novicida* covered with GBPs is targeted by immunity‐related GTPase B10 (IRGB10) with further activation of AIM2.^133^ IRGB10 was also shown to contribute towards activation of the caspase‐11–NLRP3 inflammasome in response to Gram‐negative bacteria, other than *F. novicida*.^133^

## IFI16

In a similar manner to AIM2, IFNγ‐inducible protein‐16 (IFI16) is an intracellular innate sensor that detects viral RNA, single and double‐stranded viral DNA.^134^, ^135^, ^136^, ^137^ AIM2 and IFI16 are members of the pyrin and HIN domain (PYHIN) protein family, and these two are also known as ALRs.^135^, ^138^ IFI16 has been shown to detect herpes simplex virus type 1 (HSV‐1),^135^ Kaposi sarcoma‐associated herpesvirus,^139^ vaccinia virus,^140^ HIV,^134^ chikungunya virus^141^ and influenza A virus.^136^ Unlike AIM2, IFI16 contains two HIN‐200 subunits, HINa and HINb, that detect the nucleic acid in question,^135^ or enhance the transcription of retinoic acid‐inducible gene‐I (RIG‐I).^136^, ^137^

Activation of IFI16 through sensing nucleic acids induces activation of the STING‐TBK1 pathway, which leads either to the induction of type I IFNs through IRF3,^142^ IL‐1β secretion by interactions of its pyrin domain that interacts with caspase‐1 and ASC to form a functional inflammasome,^139^ or NF‐κB activation after nuclear DNA damage.^143^ IFI16 has also been associated with cervical cancer progression by inducing the immune checkpoint programmed cell death 1 ligand 1 (PD‐L1) via the STING/TBK1/NF‐κB pathway.^144^ This could be of high importance because of the immunomodulatory effects of PD‐L1 in several cancers.^145^, ^146^ Regarding inflammatory‐related disorders, the serum levels of IFI16, as well as anti‐IFI16 antibodies, were elevated in psoriatic arthritis (PsA), which was more pronounced in patients with high CRP levels.^147^ Moreover, STING also negatively regulates IFI16 by recruiting the E3 ligase TRIM21, which degrades IFI16 through ubiquitin‐mediated proteasomal degradation, thus preventing IFN‐I overproduction.^148^ IFN regulation is a fundamental cellular mechanism to avoid autoinflammation, and a transcript isoform of IFI16 (IFI16‐β), inhibits the formation of the AIM2‐ASC complex.^149^ IFI16 is a key sensor of viral nucleic acids, in regulating IFN and modulating the inflammatory response.

## COPs and POPs

CARD‐only proteins (COPs) and pyrin‐only proteins (POPs) are small, cytoplasmic endogenous regulators of inflammasome assembly which are present in humans but absent in the mouse and rat genome.^150^

Pyrin‐only proteins consist of the PYD alone and are named POP1, POP2, POP3 and POP4. Expression of POP1 and POP2 is induced by several hours of TLR activation,^151^, ^152^ following which they interact with the PYD of ASC,^153^, ^154^, ^155^ seemingly binding competitively with this domain to prevent ASC oligomerisation, IL‐1β and IL‐18 release and pyroptosis.^152^, ^156^, ^157^, ^158^ Silencing of POP1 in human macrophages is reported to exacerbate inflammation, whereas POP1 expression had a robust anti‐inflammatory effect in mice.^151^ POP2 has additionally been shown to inhibit NF‐κB^152^, ^154^, ^155^, ^158^; however, elevated IFNγ levels were seen in POP2‐expressing transgenic mice, suggesting a mechanism by which POP2 heightens immune responses.^158^ POP3 differs in that it binds to the PYDs of the DNA sensors AIM2 and IFI16, but not of ASC,^159^ and is stimulated by dsDNA viruses.^159^ POP3 appears not to affect NF‐κB and MAPK signalling or inflammatory cytokine release in response to NLR inflammasome agonists, but instead selectively inhibit AIM2‐like receptor (ALR) inflammasomes and their downstream inflammatory processes.^159^ Finally, POP4 is the most recently discovered POP whose expression is upregulated by LPS stimulation.^160^ It inhibits NF‐κB activity following TNF stimulation by blocking TLR‐induced RelA/p65 transactivation and secretion of NF‐κB‐regulated cytokines, such as TNF and IL‐6.^160^

CARD‐only proteins, in contrast, consist of a CARD domain and comprise CARD16 (pseudo‐IL‐1β converting enzyme (pseudo‐ICE)/Cop), CARD17 (Inca), and CARD18 (Iceberg). The first of these, CARD16, binds competitively with the caspase‐1 CARD, preventing its dimerisation and reducing the maturation and release of IL‐1β.^161^, ^162^, ^163^ It also interacts with the ASC CARD^164^ and may affect NF‐κB activation via the CARD‐containing kinase receptor‐interacting‐serine/threonine‐protein kinase 2 (RIP2).^161^, ^162^ Nevertheless, these studies were mainly carried out in cell lines in which CARD16 was transiently overexpressed,^164^ further investigation in primary cells is required to support the physiological relevance of these studies. Similarly, CARD17 interacts with itself to form dimers, also binding to other COPs and the CARD of caspase‐1.^162^, ^165^ Its expression is upregulated by pro‐inflammatory stimuli, in particular IFNγ.^162^ The proposed mechanism of action of CARD17 is that it binds specifically to the filamentous form of caspase‐1, localising at the tip of caspase‐1 CARD filaments to act as a cap, thereby preventing caspase‐1 CARD polymerisation and subsequent inflammasome activity.^165^ In contrast to CARD16, CARD17 does not affect TNF‐induced NF‐κB activation,^162^, ^165^ apparently specifically targeting caspase‐1 activation. CARD18 interacts with the pro‐caspase‐1 CARD, as well as self‐aggregating,^161^, ^165^, ^166^ although the evidence regarding its function is conflicting. CARD18 has been implicated as acting in a negative feedback mechanism to inhibit RIP2 binding to caspase‐1 and disrupting existing RIP2/caspase‐1 complex to reduce IL‐1β release.^161^, ^166^ However, one study found that CARD18 did not inhibit inflammasome activation, despite being incorporated into caspase‐1.^165^ This may be because CARD18 expression is upregulated by LPS or TNF stimulation over several hours, suggesting this protein is produced slowly so that the beneficial effects of inflammation can occur before the process is halted.^166^ Further studies in relevant cellular models are required to determine the role of this protein.

## Non‐canonical inflammasome

The one‐step non‐canonical inflammasome senses the cytosolic presence of LPS derived from Gram‐negative bacteria, specifically by monocytes.^167^ LPS bind directly to caspase‐4 (caspase‐11 in mice) and caspase‐5, which initiates NLRP3‐caspase‐1‐dependent IL‐18 and IL‐1β cleavage and secretion, as well as driving inflammatory cell death via GSDMD‐dependent pyroptosis.^168^, ^169^ This mechanism of inflammasome activation was first reported by Kayagaki *et al.* in 2011 using genetic depletion of caspase‐11 in C57BL/6 mice.^2^ The presence of a mutation in caspase‐11 exon 5 in mice, resulted in failure to induce macrophage cell death and the absence of IL‐18 and IL‐1β in the serum upon *E. coli*, *C. rodentium* and *V. cholerae* infection. This study also elucidated the role of caspase‐11 in the induction of lethal septic shock in mice.

Aberrant activation of non‐canonical inflammasome factors has been associated with a range inflammatory pathologies; for instance, a case of a 10‐year‐old girl diagnosed with Aicardi‐Goutières syndrome (AGS), positive for heterozygous IFIH1 mutation (c.2336G > A (p.R779H)). The patient showed elevated histological levels of caspase‐4 and caspase‐5 processing in intestinal tissue.^170^ This was correlated with symptoms such as diarrhoea, inflammatory cell infiltration and increased levels of IL‐6 and IFNα in serum, underlining the importance of the non‐canonical inflammasome in clinical development of this case.

A Finnish family, presenting a gain‐of‐function mutation (Arg219His) in the late myeloid differentiation factor CCAAT enhancer‐binding protein epsilon (C/EBPe), developed an autosomal recessive neutrophil‐specific granule deficiency.^171^ Clinically, these patients present symptoms related with autoinflammation, immunodeficiency and neutrophil failure. Whole‐transcriptome and qPCR screening of patients' granulocytes showed upregulation of PRTN3, a regulator of both IL‐1β and IL‐18. Canonical (NLRP3, IL‐18) and non‐canonical inflammasome elements (CASP5) were transcriptionally upregulated in macrophages.^171^ Indeed, histological examination of LPS‐stimulated macrophages derived from these patients revealed a unique signal of caspase‐5 but not caspase‐1 cleavage, compared with healthy controls. This study supports the functional relevance of the non‐canonical inflammasome in autoinflammatory diseases.

Other studies have highlighted an important role of the non‐canonical inflammasome in senescence biology. The senescence‐associated secretory phenotype (SASP) is partly controlled by activation of the non‐canonical inflammasome *in vitro*.^172^ Genetic depletion of caspase‐5 reduced the secretion of hallmark cytokine factors related to SASP, such as of IL‐1α, IL‐6, IL‐8 and MCP‐1 factors in fibroblasts.^173^ In a different study, augmented expression levels and activation of caspase‐4, via cytosolic LPS, induced a senescence state and pyroptotic cell death in IMR‐90 fibroblasts.^174^ This was reflected by raised levels of p16INK4a, p21CIP1 proteins, and increased activity of senescence‐associated‐β‐galactosidase. Moreover, *in vivo* studies using NF‐κB KO mice showed a correlation in the expression levels of caspase‐11 with p21 in lung epithelial cells. Finally, caspase‐11 expression also correlated with loci of telomere‐associated DNA‐damage response, a hallmark of senescence, in alveolar cells of 24‐month aged mice. Overall, these studies support the notion that induction of non‐canonical inflammasome pathways is relevant to the biology of senescence and may present a viable therapeutic opportunity for a variety age‐related diseases.

## Inflammasomes in autoinflammatory disorders

### TRAPS

TRAPS is a rare monogenic autosomal dominant autoinflammatory disorder linked to mutations in TNFRSF1A.^175^ Its clinical manifestations include prolonged bouts of fever, associated with marked left‐shifted leukocytosis, skin rash, per‐orbital oedema, pleuritic chest pain and AA amyloidosis.^176^, ^177^ To date, over 100 pathogenic variants of TNFRSF1A have been reported in Infevers,^178^ and 97 of these are single‐nucleotide missense mutations within exons 2, 3 and 4, and 80% of the variants are located within the first two cysteine‐rich domains (CRD1, CRD2).

Our understanding of TRAPS pathophysiology has been hindered by the marked heterogeneity between each variant, and varying experimental conditions, such as differences in cell lines, mutant constructs for transfection and cell‐type effects.^179^ Blood soluble TNFR1 levels are reduced by about 50% in people affected by TRAPS, and this information was paramount to gene discovery.^175^ Thus, an initial hypothesis was that impaired shedding of the receptor TNFR1 resulted in reduced levels of circulating TNFR1 acting as a TNF inhibitor.^175^ But shedding anomalies could not be reproduced among all TNFR1 variants and all cell types,^179^ and further data showed that mutated TNFR1 subcellular traffic was disturbed, leading to abnormal ER retention and reduced cellular membrane expression.^179^, ^180^ Mutated ER‐retained TNFR1 self‐aggregates^180^ and probably transduces inflammatory signals on its own,^179^, ^180^, ^181^ as TRAPS patients' monocytes show ligand‐independent NF‐κB activation,^179^ particularly as mutated TNFR1 is often unable to bind TNF.^181^ TNFR1 variants also lead to increased mitochondrial ROS (mtROS) production, a known activator of the NLRP3 inflammasome.^182^ Inhibition of mtROS impedes pro‐inflammatory cytokine production in mouse embryonic fibroblasts transfected with TNFRSF1A pathogenic variants.^182^ Mutated variants of TNFR1 also induce atypical ER stress and unfolded protein response (UPR) transcriptomic signature.^183^ This is likely to result in NLRP3 activation, as ER‐retained TNFR1 can recruit the ER stress sensor, IRE1α, an essential UPR effector^184^ whose inhibition prevents NLRP3 activation.^185^, ^186^ Indeed, IRE1α can promote NLRP3 inflammasome assembly by damaging mitochondria via caspase‐2 and Bid activation.^185^

These molecular elements correlate quite well with therapeutics. Indeed, TNF blockade is inconsistently efficient in TRAPS,^187^ as endocellular TNFR1 aberrantly signals independently of its ligand. Meanwhile, IL‐1 blockade is very effective in producing clinical remission in selected patients,^177^, ^188^ which suggests that the NLRP3 inflammasome is responsible for proinflammatory cytokine secretion in TRAPS. If confirmed, NLRP3 inhibitors, such as MCC950^189^ and OLT1177,^190^ could be candidates for tailored treatment in TRAPS.

### CAPS/NLRP3‐AIDs

Cryopyrin‐associated periodic syndromes also known as NLRP3‐associated autoinflammatory diseases (NLRP3‐AIDs) comprise a series of rare monogenic autoinflammatory conditions which range in severity from the mildest FCAS, to the moderate Muckle–Wells syndrome (MWS), and more severe CINCA/NOMID.^191^ These disorders are caused by gain‐of‐function mutations in the *NLRP3* gene, with almost 100 known pathogenic mutations.^192^ CAPS‐related mutations are associated with systemic inflammation, which may lead to irreversible organ damage if untreated, and symptoms including urticaria‐like rash, cold‐induced fever and sensorineural hearing loss.^191^

The pathogenesis of these disorders stems from the constitutive activation of the NLRP3 inflammasome and associated increase in caspase‐1 activity, IL‐1β and IL‐18 release and pyroptosis.^193^, ^194^ Monocytes and macrophages from CAPS patients release a constitutively high level of IL‐1β compared with healthy controls,^195^, ^196^, ^197^ with IL‐1β secretion occurring much faster in monocytes from CAPS patients.^198^ The NLRP3 inflammasome in monocytes from FCAS patients can also be activated by mildly hypothermic temperatures of 32°C.^199^ Monocytes from CAPS patients and DCs from mice harbouring CAPS‐related mutations also produce elevated IL‐18, and mouse models of CAPS have shown that IL‐1β and IL‐18 drive the pathology at different disease stages, with IL‐18 contributing to early inflammation and IL‐1β to later systemic inflammation.^200^ Furthermore, significant inflammation was still observed when these mice were bred onto an IL‐1 receptor (IL‐1R) and IL‐18 receptor (IL‐18R)‐deficient background, indicating that inflammation is not fully dependent on these cytokines in mice. In contrast, caspase‐1 and GSDMD are both required for CAPS pathogenesis in mice, as knockout of each of these proteins prevented autoinflammatory symptoms in murine models.^197^, ^200^, ^201^, ^202^ Although further research is required in human studies, at this stage, it would seem that CAPS pathogenesis is primarily dependent on caspase‐1 and GSDMD, and on hypersecretion IL‐1β and IL‐18 to a lesser extent.

The three IL‐1 inhibitors currently used in clinical practice to treat CAPS are anakinra (Kineret), a recombinant version of the human IL‐1R antagonist protein (IL‐1Ra) canakinumab (Ilaris), a human monoclonal antibody which targets IL‐1β, and rilonacept (Arcalyst), a fusion of the human IL‐1R component (IL‐1R1) and IL‐1R accessory‐protein (IL‐1RAcP) which potently binds IL‐1β and IL‐1α.^203^ Potential future therapeutics are also emerging, including two small‐molecule NLRP3 inhibitors related to the well‐characterised MCC950,^204^ Inzomelid (clinical trial identifiers NCT04015076 and NCT04086602) and Somalix. These inhibitors have completed phase 1 trials, with Inzomelid completing phase 1b in CAPS patients.^205^, ^206^ Further potential treatments which have completed phase 1 clinical trials include the tryptophan analogue, Tranilast (NCT03923140), a novel hybrid Fc‐fused IL‐1Ra (NCT02175056),^207^ and ATI‐450, a small‐molecule inhibitor of the p38α mitogen‐activated protein kinase (MAPK)/MAPK‐activated protein kinase 2 (MK2) inflammatory signalling pathway,^208^ although the COVID‐19 pandemic has hindered recruitment for phase 2 trials.

### FMF and PAAND

FMF is a monogenic autosomal recessive autoinflammatory disorder that was first described clinically in the late 1940s.^209^, ^210^ Patients with FMF suffer from recurring bouts of fever, abdominal or chest pain, arthritis, pseudo‐erysipelas and they may also be afflicted with AA amyloidosis and mesothelioma. The disease was linked, in 1997, to mutations in the MEFV gene corresponding to the pyrin protein^211^, ^212^ (Figure 3).

Mutations in patients with FMF generally involve the C‐terminal B30.2 domain of pyrin. This domain does not exist in rodents, imposing a significant caveat on the extrapolation of data from murine models.^213^ Mutations in FMF do not impede the interaction of pyrin with caspase‐1 and pro‐IL‐1β^214^; however, they substantially decrease the binding of pyrin to protein kinases N (PKN) 1 and 2.^93^ PKNs are kinases belonging to the protein kinase C superfamily and also RhoA GTPase effectors. These enzymes phosphorylate pyrin on serines‐208 and ‐242, which enables 14–3‐3 protein to bind and inhibit pyrin activation.^93^ Thus, inactivation of RhoA triggers inflammation by removing pyrin inhibition by PKN‐dependent phosphorylation and FMF mutations may decrease PKN‐mediated inhibition of pyrin.^93^

Colchicine, which inhibits microtubular polymerisation,^214^ remains the cornerstone of FMF treatment since the 1970s.^215^ In healthy monocytes, pyrin activation depends on both serines‐208 and ‐242 dephosphorylation and efficient microtubule assembly.^99^, ^216^ Colchicine inhibits this latter step without affecting the former; however, colchicine is less effective *in vitro* as an inhibitor of dephosphorylation‐dependent pyrin activation in FMF monocytes than that in HC monocytes.^99^

Of note, an autosomal dominant disorder termed pyrin‐associated autoinflammation with neutrophilic dermatosis (PAAND), clinically different from FMF, is caused by specific mutations in pyrin that abolish the 14–3‐3 binding motif.^217^, ^218^ Interestingly, FMF mutations do not seem to affect 14–3‐3 binding.^217^ Serum levels of proinflammatory cytokines may be tenfold higher in FMF than in PAAND,^99^, ^217^, ^218^ implying that additional control mechanisms, distinct from 14–3‐3 binding, may be intact in PAAND but absent in FMF.

Mutations in *GSDMA, Gsdma3, GSDMB* and *GSDME* genes are associated with asthma, systemic sclerosis, alopecia, hearing loss and cancer^219^; while mutations in *GSDMD* are generally associated with inflammation in certain non‐infectious conditions, normally driven by pyroptosis.^219^ GSDMD has been linked with several non‐infectious human diseases, such as autoimmune encephalomyelitis, Kawasaki disease, kidney disease, FMF and NOMID/CINCA where inflammation plays a crucial role in the pathophysiology of the disease.^219^, ^220^, ^221^ Ablation of *Gsdmd* in FMF and NOMID/CINCA mouse models protected the animals from the inflammatory‐related symptoms normally presented during these conditions.^220^, ^221^

Anti‐IL‐1 inhibitors acting downstream of inflammasome activation, are a second‐line treatment for FMF,^215^, ^222^ and the treatment of choice in PAAND.^14^, ^217^ Anti‐TNF inhibitors could be useful in certain patients with articular phenotypes, while small‐molecule caspase‐1 inhibitors could theoretically target FMF autoinflammation.

## Final remarks

Molecular characterisation of inflammasomes has revolutionised our understanding of several immune‐mediated inflammatory disorders. It is clear that inflammasomes are key protectors of tissues from pathogens, but, nevertheless, when these mechanisms are dysregulated, this can lead to autoinflammation. Inflammasome activity requires tight regulation to avoid chronic inflammation, and, as seen in several autoinflammatory disorders, the smallest modification in protein composition may lead to autoinflammation. Understating these processes has led us to the development of several inhibitors which are essential to more effective therapeutics. Although our understanding of the intrinsic mechanisms underlying these disorders has grown exponentially, several questions remain regarding the pathogenesis of these disorders.

## Conflict of Interest

The authors declare that the research was conducted in the absence of any commercial or financial relationships that could be construed as a potential conflict of interest.

## AUTHOR CONTRIBUTIONS

**Samuel Lara‐Reyna:** Conceptualization; funding acquisition; visualization; writing – original draft; writing – review and editing. **Emily A Caseley:** Writing – original draft; writing – review and editing. **Joanne Topping:** Writing – original draft; writing – review and editing. **François Rodrigues:** Writing – original draft; writing – review and editing. **Jorge Jimenez Macias:** Writing – original draft; writing – review and editing. **Sean E Lawler:** Writing – original draft; writing – review and editing. **Michael F McDermott:** Conceptualization; funding acquisition; writing – original draft; writing – review and editing.

## References

[1] Thaiss CA , Levy M , Itav S , Elinav E . Integration of innate immune signaling. Trends Immunol 2016; 37: 84–101. 26755064 10.1016/j.it.2015.12.003

[2] Martinon F , Burns K , Tschopp J . The inflammasome: a molecular platform triggering activation of inflammatory caspases and processing of proIL‐beta. Mol Cell 2002; 10: 417–426. 12191486 10.1016/s1097-2765(02)00599-3

[3] Guo H , Callaway JB , Ting JP . Inflammasomes: mechanism of action, role in disease, and therapeutics. Nat Med 2015; 21: 677–687. 26121197 10.1038/nm.3893 PMC4519035

[4] Broz P , Dixit VM . Inflammasomes: mechanism of assembly, regulation and signalling. Nat Rev Immunol 2016; 16: 407–420. 27291964 10.1038/nri.2016.58

[5] Sharma D , Malik A , Guy C , Vogel P , Kanneganti TD . TNF/TNFR axis promotes pyrin inflammasome activation and distinctly modulates pyrin inflammasomopathy. J Clin Invest 2019; 129: 150–162. 30457980 10.1172/JCI121372 PMC6307946

[6] Zheng D , Liwinski T , Elinav E . Inflammasome activation and regulation: toward a better understanding of complex mechanisms. Cell Discov 2020; 6: 36. 32550001 10.1038/s41421-020-0167-x PMC7280307

[7] Wittmann M , Kingsbury SR , McDermott MF . Is caspase 1 central to activation of interleukin‐1? Joint Bone Spine 2011; 78: 327–330. 21459652 10.1016/j.jbspin.2011.02.004

[8] Masumoto J , Taniguchi S , Sagara J . Pyrin N‐terminal homology domain‐ and caspase recruitment domain‐dependent oligomerization of ASC. Biochem Biophys Res Commun 2001; 280: 652–655. 11162571 10.1006/bbrc.2000.4190

[9] Franchi L , Eigenbrod T , Munoz‐Planillo R , Nunez G . The inflammasome: a caspase‐1‐activation platform that regulates immune responses and disease pathogenesis. Nat Immunol 2009; 10: 241–247. 19221555 10.1038/ni.1703 PMC2820724

[10] Downs KP , Nguyen H , Dorfleutner A , Stehlik C . An overview of the non‐canonical inflammasome. Mol Asp Med 2020; 76: e100924. 10.1016/j.mam.2020.100924 PMC7808250 33187725

[11] Yi YS . Caspase‐11 non‐canonical inflammasome: a critical sensor of intracellular lipopolysaccharide in macrophage‐mediated inflammatory responses. Immunology 2017; 152: 207–217. 28695629 10.1111/imm.12787 PMC5588777

[12] Devi S , Stehlik C , Dorfleutner A . An update on CARD only proteins (COPs) and PYD only proteins (POPs) as inflammasome regulators. Int J Mol Sci 2020; 21: e6901. 32962268 10.3390/ijms21186901 PMC7555848

[13] Wilson SP , Cassel SL . Inflammasome‐mediated autoinflammatory disorders. Postgrad Med 2010; 122: 125–133. 10.3810/pgm.2010.09.2209 PMC3648991 20861596

[14] Savic S , Caseley EA , McDermott MF . Moving towards a systems‐based classification of innate immune‐mediated diseases. Nat Rev Rheumatol 2020; 16: 222–237. 32107482 10.1038/s41584-020-0377-5

[15] Fernandes FP , Leal VNC , Souza de Lima D , Reis EC , Pontillo A . Inflammasome genetics and complex diseases: a comprehensive review. Eur J Hum Genet 2020; 28: 1307–1321. 32499599 10.1038/s41431-020-0631-y PMC7608315

[16] Swanson KV , Deng M , Ting JP . The NLRP3 inflammasome: molecular activation and regulation to therapeutics. Nat Rev Immunol 2019; 19: 477–489. 31036962 10.1038/s41577-019-0165-0 PMC7807242

[17] Seok JK , Kang HC , Cho YY , Lee HS , Lee JY . Regulation of the NLRP3 inflammasome by post‐translational modifications and small molecules. Front Immunol 2020; 11: 618231. 33603747 10.3389/fimmu.2020.618231 PMC7884467

[18] Samir P , Malireddi RKS , Kanneganti TD . The PANoptosome: A deadly protein complex driving pyroptosis, apoptosis, and necroptosis (PANoptosis). Front Cell Infect Microbiol 2020; 10: 238. 32582562 10.3389/fcimb.2020.00238 PMC7283380

[19] Shi J , Zhao Y , Wang K *et al*. Cleavage of GSDMD by inflammatory caspases determines pyroptotic cell death. Nature 2015; 526: 660–665. 26375003 10.1038/nature15514

[20] He WT , Wan H , Hu L *et al*. Gasdermin D is an executor of pyroptosis and required for interleukin‐1β secretion. Cell Res 2015; 25: 1285–1298. 26611636 10.1038/cr.2015.139 PMC4670995

[21] Van Opdenbosch N , Gurung P , Vande Walle L , Fossoul A , Kanneganti TD , Lamkanfi M . Activation of the NLRP1b inflammasome independently of ASC‐mediated caspase‐1 autoproteolysis and speck formation. Nat Commun 2014; 5: 3209. 24492532 10.1038/ncomms4209 PMC3926011

[22] Taabazuing CY , Griswold AR , Bachovchin DA . The NLRP1 and CARD8 inflammasomes. Immunol Rev 2020; 297: 13–25. 32558991 10.1111/imr.12884 PMC7483925

[23] Duncan JA , Canna SW . The NLRC4 Inflammasome. Immunol Rev 2018; 281: 115–123. 29247997 10.1111/imr.12607 PMC5897049

[24] Bachovchin DA . NLRP1: a jack of all trades, or a master of one? Mol Cell 2021; 81: 423–425. 33545058 10.1016/j.molcel.2021.01.001

[25] Tschopp J , Martinon F , Burns K . NALPs: a novel protein family involved in inflammation. Nat Rev Mol Cell Biol 2003; 4: 95–104. 12563287 10.1038/nrm1019

[26] D'Osualdo A , Weichenberger CX , Wagner RN , Godzik A , Wooley J , Reed JC . CARD8 and NLRP1 Undergo Autoproteolytic Processing through a ZU5‐Like Domain. PLoS One 2011; 6: e27396. 22087307 10.1371/journal.pone.0027396 PMC3210808

[27] Zhong FL , Robinson K , Teo DET *et al*. Human DPP9 represses NLRP1 inflammasome and protects against autoinflammatory diseases via both peptidase activity and FIIND domain binding. J Biol Chem 2018; 293: 18864–18878. 30291141 10.1074/jbc.RA118.004350 PMC6295727

[28] Huang M , Zhang X , Toh GA *et al*. Structural and biochemical mechanisms of NLRP1 inhibition by DPP9. Nature 2021; 592: 773–777. 33731929 10.1038/s41586-021-03320-w PMC8081665

[29] Moecking J , Laohamonthonkul P , Chalker K *et al*. NLRP1 variant M1184V decreases inflammasome activation in the context of DPP9 inhibition and asthma severity. J Allergy Clin Immunol 2021; 147: 2134, e2120–2145. 33378691 10.1016/j.jaci.2020.12.636 PMC8168955

[30] Zhong FL , Mamai O , Sborgi L *et al*. Germline NLRP1 mutations cause skin inflammatory and cancer susceptibility syndromes via inflammasome activation. Cell 2016; 167: 187, e117–202. 27662089 10.1016/j.cell.2016.09.001

[31] Jin Y , Mailloux CM , Gowan K *et al*. NALP1 in vitiligo‐associated multiple autoimmune disease. N Engl J Med 2007; 356: 1216–1225. 17377159 10.1056/NEJMoa061592

[32] Drutman SB , Haerynck F , Zhong FL *et al*. Homozygous NLRP1 gain‐of‐function mutation in siblings with a syndromic form of recurrent respiratory papillomatosis. Proc Natl Acad Sci USA 2019; 116: 19055–19063. 31484767 10.1073/pnas.1906184116 PMC6754618

[33] Linder A , Bauernfried S , Cheng Y *et al*. CARD8 inflammasome activation triggers pyroptosis in human T cells. EMBO J 2020; 39: e105071. 32840892 10.15252/embj.2020105071 PMC7527815

[34] Wang Q , Gao H , Clark KM *et al*. CARD8 is an inflammasome sensor for HIV‐1 protease activity. Science 2021; 371: eabe1707. 33542150 10.1126/science.abe1707 PMC8029496

[35] Rao SD , Chen Q , Wang Q *et al*. M24B aminopeptidase inhibitors selectively activate the CARD8 inflammasome. Nat Chem Biol 2022; 18: 565–574. 35165443 10.1038/s41589-021-00964-7 PMC9179932

[36] Robinson KS , Teo DET , Tan KS *et al*. Enteroviral 3C protease activates the human NLRP1 inflammasome in airway epithelia. Science 2020; 370: eaay2002. 33093214 10.1126/science.aay2002

[37] Tsu BV , Beierschmitt C , Ryan AP , Agarwal R , Mitchell PS , Daugherty MD . Diverse viral proteases activate the NLRP1 inflammasome. elife 2021; 10: e60609. 33410748 10.7554/eLife.60609 PMC7857732

[38] Bauernfried S , Scherr MJ , Pichlmair A , Duderstadt KE , Hornung V . Human NLRP1 is a sensor for double‐stranded RNA. Science 2021; 371: eabd0811. 33243852 10.1126/science.abd0811

[39] Chui AJ , Okondo MC , Rao SD *et al*. N‐terminal degradation activates the NLRP1B inflammasome. Science 2019; 364: 82–85. 30872531 10.1126/science.aau1208 PMC6610862

[40] Sasaki Y , Otsuka K , Arimochi H , Tsukumo SI , Yasutomo K . Distinct roles of IL‐1β and IL‐18 in NLRC4‐induced autoinflammation. Front Immunol 2020; 11: 591713. 33178225 10.3389/fimmu.2020.591713 PMC7592392

[41] Brydges SD , Broderick L , McGeough MD , Pena CA , Mueller JL , Hoffman HM . Divergence of IL‐1, IL‐18, and cell death in NLRP3 inflammasomopathies. J Clin Invest 2013; 123: 4695–4705. 24084736 10.1172/JCI71543 PMC3809806

[42] Ge Q , Chen X , Zhao Y , Mu H , Zhang J . Modulatory mechanisms of NLRP3: Potential roles in inflammasome activation. Life Sci 2021; 267: 118918. 33352170 10.1016/j.lfs.2020.118918

[43] Kelley N , Jeltema D , Duan Y , He Y . The NLRP3 inflammasome: an overview of mechanisms of activation and regulation. Int J Mol Sci 2019; 20: 3328. 10.3390/ijms20133328 PMC6651423 31284572

[44] Wang Z , Zhang S , Xiao Y *et al*. NLRP3 inflammasome and inflammatory diseases. Oxidative Med Cell Longev 2020; 2020: 4063562–4063511. 10.1155/2020/4063562 PMC7049400 32148650

[45] Gritsenko A , Yu S , Martin‐Sanchez F *et al*. Priming is dispensable for NLRP3 inflammasome activation in human monocytes in vitro. Front Immunol 2020; 11: 565924. 33101286 10.3389/fimmu.2020.565924 PMC7555430

[46] Bezbradica JS , Coll RC , Schroder K . Sterile signals generate weaker and delayed macrophage NLRP3 inflammasome responses relative to microbial signals. Cell Mol Immunol 2017; 14: 118–126. 26996064 10.1038/cmi.2016.11 PMC5214936

[47] Duan Y , Zhang L , Angosto‐Bazarra D , Pelegrin P , Nunez G , He Y . RACK1 mediates NLRP3 inflammasome activation by promoting NLRP3 active conformation and inflammasome assembly. Cell Rep 2020; 33: 108405. 33207200 10.1016/j.celrep.2020.108405 PMC7709964

[48] Ross C , Chan AH , von Pein JB , Maddugoda MP , Boucher D , Schroder K . Inflammatory caspases: toward a unified model for caspase activation by inflammasomes. Annu Rev Immunol 2022; 40: 249–269. 35080918 10.1146/annurev-immunol-101220-030653

[49] Caseley EA , Poulter JA , Rodrigues F , Immunome Project Consortium for Autoinflammatory D , MF MD . Inflammasome inhibition under physiological and pharmacological conditions. Genes Immun 2020; 21: 211–223. 32681062 10.1038/s41435-020-0104-x

[50] Zhang X , Xu A , Lv J *et al*. Development of small molecule inhibitors targeting NLRP3 inflammasome pathway for inflammatory diseases. Eur J Med Chem 2020; 185: 111822. 31699536 10.1016/j.ejmech.2019.111822

[51] Wang W , Hu D , Wu C *et al*. STING promotes NLRP3 localization in ER and facilitates NLRP3 deubiquitination to activate the inflammasome upon HSV‐1 infection. PLoS Pathog 2020; 16: e1008335. 32187211 10.1371/journal.ppat.1008335 PMC7080238

[52] Hooftman A , Angiari S , Hester S *et al*. The immunomodulatory metabolite itaconate modifies NLRP3 and inhibits inflammasome activation. Cell Metab 2020; 32: 468, e467–478. 32791101 10.1016/j.cmet.2020.07.016 PMC7422798

[53] Mills EL , Ryan DG , Prag HA *et al*. Itaconate is an anti‐inflammatory metabolite that activates Nrf2 via alkylation of KEAP1. Nature 2018; 556: 113–117. 29590092 10.1038/nature25986 PMC6047741

[54] Dufies O , Doye A , Courjon J *et al*. *Escherichia coli* Rho GTPase‐activating toxin CNF1 mediates NLRP3 inflammasome activation via p21‐activated kinases‐1/2 during bacteraemia in mice. Nat Microbiol 2021; 6: 401–412. 33432150 10.1038/s41564-020-00832-5 PMC7116836

[55] Niu T , De Rosny C , Chautard S *et al*. NLRP3 phosphorylation in its LRR domain critically regulates inflammasome assembly. Nat Commun 2021; 12: 5862. 34615873 10.1038/s41467-021-26142-w PMC8494922

[56] Gross O , Thomas CJ , Guarda G , Tschopp J . The inflammasome: an integrated view. Immunol Rev 2011; 243: 136–151. 21884173 10.1111/j.1600-065X.2011.01046.x

[57] Latz E , Xiao TS , Stutz A . Activation and regulation of the inflammasomes. Nat Rev Immunol 2013; 13: 397–411. 23702978 10.1038/nri3452 PMC3807999

[58] Shimada K , Crother TR , Karlin J *et al*. Oxidized mitochondrial DNA activates the NLRP3 inflammasome during apoptosis. Immunity 2012; 36: 401–414. 22342844 10.1016/j.immuni.2012.01.009 PMC3312986

[59] Zhou R , Yazdi AS , Menu P , Tschopp J . A role for mitochondria in NLRP3 inflammasome activation. Nature 2011; 469: 221–225. 21124315 10.1038/nature09663

[60] Bauernfeind F , Bartok E , Rieger A , Franchi L , Nunez G , Hornung V . Cutting edge: reactive oxygen species inhibitors block priming, but not activation, of the NLRP3 inflammasome. J Immunol 2011; 187: 613–617. 21677136 10.4049/jimmunol.1100613 PMC3131480

[61] Zhong Z , Liang S , Sanchez‐Lopez E *et al*. New mitochondrial DNA synthesis enables NLRP3 inflammasome activation. Nature 2018; 560: 198–203. 30046112 10.1038/s41586-018-0372-z PMC6329306

[62] Tschopp J , Schroder K . NLRP3 inflammasome activation: The convergence of multiple signalling pathways on ROS production? Nat Rev Immunol 2010; 10: 210–215. 20168318 10.1038/nri2725

[63] Scozzi D , Cano M , Ma L *et al*. Circulating mitochondrial DNA is an early indicator of severe illness and mortality from COVID‐19. JCI Insight 2021; 6: e143299. 10.1172/jci.insight.143299 PMC7934921 33444289

[64] Nieto‐Torres JL , Verdia‐Baguena C , Jimenez‐Guardeno JM *et al*. Severe acute respiratory syndrome coronavirus E protein transports calcium ions and activates the NLRP3 inflammasome. Virology 2015; 485: 330–339. 26331680 10.1016/j.virol.2015.08.010 PMC4619128

[65] Chen IY , Moriyama M , Chang MF , Ichinohe T . severe acute respiratory syndrome coronavirus viroporin 3a activates the NLRP3 inflammasome. Front Microbiol 2019; 10: 50. 30761102 10.3389/fmicb.2019.00050 PMC6361828

[66] Pan P , Shen M , Yu Z *et al*. SARS‐CoV‐2 N protein promotes NLRP3 inflammasome activation to induce hyperinflammation. Nat Commun 2021; 12: 4664. 34341353 10.1038/s41467-021-25015-6 PMC8329225

[67] Kim BH , Chee JD , Bradfield CJ , Park ES , Kumar P , MacMicking JD . Interferon‐induced guanylate‐binding proteins in inflammasome activation and host defense. Nat Immunol 2016; 17: 481–489. 27092805 10.1038/ni.3440 PMC4961213

[68] Tretina K , Park ES , Maminska A , MacMicking JD . Interferon‐induced guanylate‐binding proteins: Guardians of host defense in health and disease. J Exp Med 2019; 216: 482–500. 30755454 10.1084/jem.20182031 PMC6400534

[69] Shenoy AR , Wellington DA , Kumar P *et al*. GBP5 promotes NLRP3 inflammasome assembly and immunity in mammals. Science 2012; 336: 481–485. 22461501 10.1126/science.1217141

[70] Ghimire L , Paudel S , Jin L , Baral P , Cai S , Jeyaseelan S . NLRP6 negatively regulates pulmonary host defense in Gram‐positive bacterial infection through modulating neutrophil recruitment and function. PLoS Pathog 2018; 14: e1007308. 30248149 10.1371/journal.ppat.1007308 PMC6171945

[71] Hara H , Seregin SS , Yang D *et al*. The NLRP6 inflammasome recognizes lipoteichoic acid and regulates gram‐positive pathogen infection. Cell 2018; 175: 1651, e1614–1664. 30392956 10.1016/j.cell.2018.09.047 PMC6294477

[72] Anand PK , Malireddi RS , Lukens JR *et al*. NLRP6 negatively regulates innate immunity and host defence against bacterial pathogens. Nature 2012; 488: 389–393. 22763455 10.1038/nature11250 PMC3422416

[73] Wlodarska M , Thaiss CA , Nowarski R *et al*. NLRP6 inflammasome orchestrates the colonic host‐microbial interface by regulating goblet cell mucus secretion. Cell 2014; 156: 1045–1059. 24581500 10.1016/j.cell.2014.01.026 PMC4017640

[74] Normand S , Delanoye‐Crespin A , Bressenot A *et al*. Nod‐like receptor pyrin domain‐containing protein 6 (NLRP6) controls epithelial self‐renewal and colorectal carcinogenesis upon injury. Proc Natl Acad Sci USA 2011; 108: 9601–9606. 21593405 10.1073/pnas.1100981108 PMC3111299

[75] Ydens E , Demon D , Lornet G *et al*. Nlrp6 promotes recovery after peripheral nerve injury independently of inflammasomes. J Neuroinflammation 2015; 12: 1–14. 10.1186/s12974-015-0367-8 PMC4528710 26253422

[76] Wang P‐F , Li Z‐G , Zhang Y *et al*. NLRP6 inflammasome ameliorates brain injury after intracerebral hemorrhage. Front Cell Neurosci 2017; 11: 206. 28798666 10.3389/fncel.2017.00206 PMC5527702

[77] Grenier JM , Wang L , Manji GA *et al*. Functional screening of five PYPAF family members identifies PYPAF5 as a novel regulator of NF‐κB and caspase‐1. FEBS Lett 2002; 530: 73–78. 12387869 10.1016/s0014-5793(02)03416-6

[78] Levy M , Thaiss CA , Zeevi D *et al*. Microbiota‐modulated metabolites shape the intestinal microenvironment by regulating NLRP6 inflammasome signaling. Cell 2015; 163: 1428–1443. 26638072 10.1016/j.cell.2015.10.048 PMC5665753

[79] Chen GY , Liu M , Wang F , Bertin J , Núñez G . A functional role for Nlrp6 in intestinal inflammation and tumorigenesis. J Immunol 2011; 186: 7187–7194. 21543645 10.4049/jimmunol.1100412 PMC3133458

[80] Elinav E , Strowig T , Kau AL *et al*. NLRP6 inflammasome regulates colonic microbial ecology and risk for colitis. Cell 2011; 145: 745–757. 21565393 10.1016/j.cell.2011.04.022 PMC3140910

[81] Levy M , Shapiro H , Thaiss CA , Elinav E . NLRP6: a multifaceted innate immune sensor. Trends Immunol 2017; 38: 248–260. 28214100 10.1016/j.it.2017.01.001

[82] Khare S , Dorfleutner A , Bryan NB *et al*. An NLRP7‐containing inflammasome mediates recognition of microbial lipopeptides in human macrophages. Immunity 2012; 36: 464–476. 22361007 10.1016/j.immuni.2012.02.001 PMC3315380

[83] Radian AD , Khare S , Chu LH , Dorfleutner A , Stehlik C . ATP binding by NLRP7 is required for inflammasome activation in response to bacterial lipopeptides. Mol Immunol 2015; 67: 294–302. 26143398 10.1016/j.molimm.2015.06.013 PMC4565763

[84] Bednash JS , Weathington N , Londino J *et al*. Targeting the deubiquitinase STAMBP inhibits NALP7 inflammasome activity. Nat Commun 2017; 8: 1–13. 28492230 10.1038/ncomms15203 PMC5437278

[85] Kinoshita T , Wang Y , Hasegawa M , Imamura R , Suda T . PYPAF3, a PYRIN‐containing APAF‐1‐like protein, is a feedback regulator of caspase‐1‐dependent interleukin‐1β secretion. J Biol Chem 2005; 280: 21720–21725. 15817483 10.1074/jbc.M410057200

[86] Messaed C , Chebaro W , Di Roberto RB *et al*. NLRP7 in the spectrum of reproductive wastage: rare non‐synonymous variants confer genetic susceptibility to recurrent reproductive wastage. J Med Genet 2011; 48: 540–548. 21659348 10.1136/jmg.2011.089144

[87] Dorfleutner A , Chu L , Stehlik C . Inhibiting the inflammasome: one domain at a time. Immunol Rev 2015; 265: 205–216. 25879295 10.1111/imr.12290 PMC4400809

[88] Messaed C , Akoury E , Djuric U *et al*. NLRP7, a nucleotide oligomerization domain‐like receptor protein, is required for normal cytokine secretion and co‐localizes with Golgi and the microtubule‐organizing center. J Biol Chem 2011; 286: 43313–43323. 22025618 10.1074/jbc.M111.306191 PMC3234874

[89] Radian AD , de Almeida L , Dorfleutner A , Stehlik C . NLRP7 and related inflammasome activating pattern recognition receptors and their function in host defense and disease. Microbes Infect 2013; 15: 630–639. 23618810 10.1016/j.micinf.2013.04.001 PMC3722249

[90] Zhou Y , Shah SZA , Yang L , Zhang Z , Zhou X , Zhao D . Virulent Mycobacterium bovis Beijing strain activates the NLRP7 inflammasome in THP‐1 macrophages. PLoS One 2016; 11: e0152853. 27043315 10.1371/journal.pone.0152853 PMC4820140

[91] Xu H , Yang JL , Gao WQ *et al*. Innate immune sensing of bacterial modifications of Rho GTPases by the Pyrin inflammasome. Nature 2014; 513: 237–241. 24919149 10.1038/nature13449

[92] Weinert C , Morger D , Djekic A , Grutter MG , Mittl PR . Crystal structure of TRIM20 C‐terminal coiled‐coil/B30.2 fragment: implications for the recognition of higher order oligomers. Sci Rep 2015; 5: 10819. 26043233 10.1038/srep10819 PMC4455283

[93] Park YH , Wood G , Kastner DL , Chae JJ . Pyrin inflammasome activation and RhoA signaling in the autoinflammatory diseases FMF and HIDS. Nat Immunol 2016; 17: 914–921. 27270401 10.1038/ni.3457 PMC4955684

[94] Waite AL , Schaner P , Hu C *et al*. Pyrin and ASC co‐localize to cellular sites that are rich in polymerizing actin. Exp Biol Med 2009; 234: 40–52. 10.3181/0806-RM-184 19109554

[95] Kim ML , Chae JJ , Park YH *et al*. Aberrant actin depolymerization triggers the pyrin inflammasome and autoinflammatory disease that is dependent on IL‐18, not IL‐1β. J Exp Med 2015; 212: 927–938. 26008898 10.1084/jem.20142384 PMC4451132

[96] Heilig R , Broz P . Function and mechanism of the pyrin inflammasome. Eur J Immunol 2018; 48: 230–238. 29148036 10.1002/eji.201746947

[97] Shohat M , Halpern GJ . Familial Mediterranean fever—A review. Genet Med 2011; 13: 487–498. 21358337 10.1097/GIM.0b013e3182060456

[98] Sharma D , Malik A , Balakrishnan A , Malireddi RKS , Kanneganti TD . RIPK3 promotes Mefv expression and pyrin inflammasome activation via modulation of mTOR signaling. J Immunol 2020; 205: 2778–2785. 32989095 10.4049/jimmunol.2000244 PMC9447007

[99] Magnotti F , Lefeuvre L , Benezech S *et al*. Pyrin dephosphorylation is sufficient to trigger inflammasome activation in familial Mediterranean fever patients. EMBO Mol Med 2019; 11: e10547. 31589380 10.15252/emmm.201910547 PMC6835204

[100] Alimov I , Menon S , Cochran N *et al*. Bile acid analogues are activators of pyrin inflammasome. J Biol Chem 2019; 294: 3359–3366. 30647128 10.1074/jbc.RA118.005103 PMC6416436

[101] Park YH , Remmers EF , Lee W *et al*. Ancient familial Mediterranean fever mutations in human pyrin and resistance to Yersinia pestis. Nat Immunol 2020; 21: 857–867. 32601469 10.1038/s41590-020-0705-6 PMC7381377

[102] Zhao Y , Yang J , Shi J *et al*. The NLRC4 inflammasome receptors for bacterial flagellin and type III secretion apparatus. Nature 2011; 477: 596–600. 21918512 10.1038/nature10510

[103] Kofoed EM , Vance RE . Innate immune recognition of bacterial ligands by NAIPs determines inflammasome specificity. Nature 2011; 477: 592–595. 21874021 10.1038/nature10394 PMC3184209

[104] Lightfield KL , Persson J , Brubaker SW *et al*. Critical function for Naip5 in inflammasome activation by a conserved carboxy‐terminal domain of flagellin. Nat Immunol 2008; 9: 1171–1178. 18724372 10.1038/ni.1646 PMC2614210

[105] Molofsky AB , Byrne BG , Whitfield NN *et al*. Cytosolic recognition of flagellin by mouse macrophages restricts *Legionella pneumophila* infection. J Exp Med 2006; 203: 1093–1104. 16606669 10.1084/jem.20051659 PMC1584282

[106] Ren T , Zamboni DS , Roy CR , Dietrich WF , Vance RE . Flagellin‐deficient *Legionella* mutants evade caspase‐1‐and Naip5‐mediated macrophage immunity. PLoS Pathog 2006; 2: e18. 16552444 10.1371/journal.ppat.0020018 PMC1401497

[107] Yang J , Zhao Y , Shi J , Shao F . Human NAIP and mouse NAIP1 recognize bacterial type III secretion needle protein for inflammasome activation. Proc Natl Acad Sci USA 2013; 110: 14408–14413. 23940371 10.1073/pnas.1306376110 PMC3761597

[108] Hu Z , Yan C , Liu P *et al*. Crystal structure of NLRC4 reveals its autoinhibition mechanism. Science 2013; 341: 172–175. 23765277 10.1126/science.1236381

[109] Li Y , Fu T‐M , Lu A *et al*. Cryo‐EM structures of ASC and NLRC4 CARD filaments reveal a unified mechanism of nucleation and activation of caspase‐1. Proc Natl Acad Sci USA 2018; 115: 10845–10852. 30279182 10.1073/pnas.1810524115 PMC6205419

[110] Miao EA , Leaf IA , Treuting PM *et al*. Caspase‐1‐induced pyroptosis is an innate immune effector mechanism against intracellular bacteria. Nat Immunol 2010; 11: 1136–1142. 21057511 10.1038/ni.1960 PMC3058225

[111] Pereira MS , Marques GG , DelLama JE , Zamboni DS . The Nlrc4 inflammasome contributes to restriction of pulmonary infection by flagellated *Legionella* spp. that trigger pyroptosis. Front Microbiol 2011; 2: 33. 21687424 10.3389/fmicb.2011.00033 PMC3109297

[112] Samir P , Malireddi R , Kanneganti T‐D . The PANoptosome: a deadly protein complex driving pyroptosis, apoptosis, and necroptosis (PANoptosis). Front Cell Infect Microbiol 2020; 10: 238. 32582562 10.3389/fcimb.2020.00238 PMC7283380

[113] Malireddi RS , Ippagunta S , Lamkanfi M , Kanneganti T‐D . Cutting edge: proteolytic inactivation of poly (ADP‐ribose) polymerase 1 by the Nlrp3 and Nlrc4 inflammasomes. J Immunol 2010; 185: 3127–3130. 20713892 10.4049/jimmunol.1001512 PMC3104018

[114] Christgen S , Zheng M , Kesavardhana S *et al*. Identification of the PANoptosome: a molecular platform triggering pyroptosis, apoptosis, and necroptosis (PANoptosis). Front Cell Infect Microbiol 2020; 10: 237. 32547960 10.3389/fcimb.2020.00237 PMC7274033

[115] Doerflinger M , Deng Y , Whitney P *et al*. Flexible usage and interconnectivity of diverse cell death pathways protect against intracellular infection. Immunity 2020; 53: 533, e537–547. 32735843 10.1016/j.immuni.2020.07.004 PMC7500851

[116] Kay C , Wang R , Kirkby M , Man SM . Molecular mechanisms activating the NAIP‐NLRC4 inflammasome: Implications in infectious disease, autoinflammation, and cancer. Immunol Rev 2020; 297: 67–82. 32729154 10.1111/imr.12906

[117] Volker‐Touw C , de Koning H , Giltay J *et al*. Erythematous nodes, urticarial rash and arthralgias in a large pedigree with NLRC4‐related autoinflammatory disease, expansion of the phenotype. Br J Dermatol 2017; 176: 244–248. 27203668 10.1111/bjd.14757

[118] Kitamura A , Sasaki Y , Abe T , Kano H , Yasutomo K . An inherited mutation in NLRC4 causes autoinflammation in human and miceAutoinflammation and mutant NLRC4. J Exp Med 2014; 211: 2385–2396. 25385754 10.1084/jem.20141091 PMC4235634

[119] Wang L , Wen W , Deng M *et al*. A novel mutation in the NBD domain of NLRC4 causes mild autoinflammation with recurrent urticaria. Front Immunol 2021; 12: 2471. 10.3389/fimmu.2021.674808 PMC8260849 34248956

[120] Sharma BR , Karki R , Kanneganti TD . Role of AIM2 inflammasome in inflammatory diseases, cancer and infection. Eur J Immunol 2019; 49: 1998–2011. 31372985 10.1002/eji.201848070 PMC7015662

[121] Kumari P , Russo AJ , Shivcharan S , Rathinam VA . AIM2 in health and disease: inflammasome and beyond. Immunol Rev 2020; 297: 83–95. 32713036 10.1111/imr.12903 PMC7668394

[122] Pierini R , Juruj C , Perret M *et al*. AIM2/ASC triggers caspase‐8‐dependent apoptosis in Francisella‐infected caspase‐1‐deficient macrophages. Cell Death Differ 2012; 19: 1709–1721. 22555457 10.1038/cdd.2012.51 PMC3438500

[123] Lammert CR , Frost EL , Bellinger CE *et al*. AIM2 inflammasome surveillance of DNA damage shapes neurodevelopment. Nature 2020; 580: 647–652. 32350463 10.1038/s41586-020-2174-3 PMC7788527

[124] Yuan B , Zhou XM , You ZQ *et al*. Inhibition of AIM2 inflammasome activation alleviates GSDMD‐induced pyroptosis in early brain injury after subarachnoid haemorrhage. Cell Death Dis 2020; 11: 76. 32001670 10.1038/s41419-020-2248-z PMC6992766

[125] Lee S , Karki R , Wang Y , Nguyen LN , Kalathur RC , Kanneganti TD . AIM2 forms a complex with pyrin and ZBP1 to drive PANoptosis and host defence. Nature 2021; 597: 415–419. 34471287 10.1038/s41586-021-03875-8 PMC8603942

[126] Gaidt MM , Ebert TS , Chauhan D *et al*. The DNA inflammasome in human myeloid cells is initiated by a STING‐cell death program upstream of NLRP3. Cell 2017; 171: 1110, e1118–1124. 29033128 10.1016/j.cell.2017.09.039 PMC5901709

[127] Hu B , Jin C , Li HB *et al*. The DNA‐sensing AIM2 inflammasome controls radiation‐induced cell death and tissue injury. Science 2016; 354: 765–768. 27846608 10.1126/science.aaf7532 PMC5640175

[128] Wu Y , Yang H , Xu S *et al*. AIM2 inflammasome contributes to aldosterone‐induced renal injury via endoplasmic reticulum stress. Clin Sci (Lond) 2022; 136: 103–120. 34935888 10.1042/CS20211075

[129] Liu L , Yang M , Kang R *et al*. HMGB1‐DNA complex‐induced autophagy limits AIM2 inflammasome activation through RAGE. Biochem Biophys Res Commun 2014; 450: 851–856. 24971542 10.1016/j.bbrc.2014.06.074 PMC4107148

[130] Liu J , Wang Y , Liao Y , Zhou Y , Zhu J . Circular RNA PPP1CC promotes Porphyromonas gingivalis‐lipopolysaccharide‐induced pyroptosis of vascular smooth muscle cells by activating the HMGB1/TLR9/AIM2 pathway. J Int Med Res 2021; 49: 300060521996564. 33769113 10.1177/0300060521996564 PMC8165858

[131] Wang J , Li R , Peng Z , Hu B , Rao X , Li J . HMGB1 participates in LPSinduced acute lung injury by activating the AIM2 inflammasome in macrophages and inducing polarization of M1 macrophages via TLR2, TLR4, and RAGE/NFκB signaling pathways. Int J Mol Med 2020; 45: 61–80. 31746367 10.3892/ijmm.2019.4402 PMC6889921

[132] Meunier E , Wallet P , Dreier RF *et al*. Guanylate‐binding proteins promote activation of the AIM2 inflammasome during infection with *Francisella novicida* . Nat Immunol 2015; 16: 476–484. 25774716 10.1038/ni.3119 PMC4568307

[133] Man SM , Karki R , Sasai M *et al*. IRGB10 liberates bacterial ligands for sensing by the AIM2 and caspase‐11‐NLRP3 inflammasomes. Cell 2016; 167: 382, e317–396. 27693356 10.1016/j.cell.2016.09.012 PMC5074697

[134] Monroe KM , Yang ZY , Johnson JR *et al*. IFI16 DNA sensor is required for death of lymphoid CD4 T cells abortively infected with HIV. Science 2014; 343: 428–432. 24356113 10.1126/science.1243640 PMC3976200

[135] Unterholzner L , Keating SE , Baran M *et al*. IFI16 is an innate immune sensor for intracellular DNA. Nat Immunol 2010; 11: 997–U942. 20890285 10.1038/ni.1932 PMC3142795

[136] Jiang Z , Wei F , Zhang Y *et al*. IFI16 directly senses viral RNA and enhances RIG‐I transcription and activation to restrict influenza virus infection. Nat Microbiol 2021; 6: 932–945. 33986530 10.1038/s41564-021-00907-x

[137] Thompson MR , Sharma S , Atianand M *et al*. Interferon γ‐inducible protein (IFI) 16 transcriptionally regulates type I interferons and other interferon‐stimulated genes and controls the interferon response to both DNA and RNA viruses. J Biol Chem 2014; 289: 23568–23581. 25002588 10.1074/jbc.M114.554147 PMC4156042

[138] Dawson MJ , Trapani JA . The interferon‐inducible autoantigen, IFI 16: localization to the nucleolus and identification of a DNA‐binding domain. Biochem Biophys Res Commun 1995; 214: 152–162. 7545391 10.1006/bbrc.1995.2269

[139] Kerur N , Veettil MV , Sharma‐Walia N *et al*. IFI16 acts as a nuclear pathogen sensor to induce the inflammasome in response to Kaposi Sarcoma‐associated herpesvirus infection. Cell Host Microbe 2011; 9: 363–375. 21575908 10.1016/j.chom.2011.04.008 PMC3113467

[140] Ansari MA , Dutta S , Veettil MV *et al*. Herpesvirus genome recognition induced acetylation of nuclear IFI16 is essential for its cytoplasmic translocation, inflammasome and IFN‐β responses. PLoS Pathog 2015; 11: e1005019. 26134128 10.1371/journal.ppat.1005019 PMC4489722

[141] Kim B , Arcos S , Rothamel K *et al*. Discovery of widespread host protein interactions with the pre‐replicated genome of CHIKV using VIR‐CLASP. Mol Cell 2020; 78: 624, e627–640. 32380061 10.1016/j.molcel.2020.04.013 PMC7263428

[142] Orzalli MH , DeLuca NA , Knipe DM . Nuclear IFI16 induction of IRF‐3 signaling during herpesviral infection and degradation of IFI16 by the viral ICP0 protein. Proc Natl Acad Sci USA 2012; 109: E3008–E3017. 23027953 10.1073/pnas.1211302109 PMC3497734

[143] Dunphy G , Flannery SM , Almine JF *et al*. Non‐canonical activation of the DNA sensing adaptor STING by ATM and IFI16 mediates NF‐κB signaling after nuclear DNA damage. Mol Cell 2018; 71: 745, e745–760. 30193098 10.1016/j.molcel.2018.07.034 PMC6127031

[144] Cai H , Yan L , Liu N , Xu M , Cai H . IFI16 promotes cervical cancer progression by upregulating PD‐L1 in immunomicroenvironment through STING‐TBK1‐NF‐kB pathway. Biomed Pharmacother 2020; 123: 109790. 31896065 10.1016/j.biopha.2019.109790

[145] Chen L , Han X . Anti‐PD‐1/PD‐L1 therapy of human cancer: past, present, and future. J Clin Invest 2015; 125: 3384–3391. 26325035 10.1172/JCI80011 PMC4588282

[146] Gou Q , Dong C , Xu H *et al*. PD‐L1 degradation pathway and immunotherapy for cancer. Cell Death Dis 2020; 11: 955. 33159034 10.1038/s41419-020-03140-2 PMC7648632

[147] De Andrea M , De Santis M , Caneparo V *et al*. Serum IFI16 and anti‐IFI16 antibodies in psoriatic arthritis. Clin Exp Immunol 2020; 199: 88–96. 31571199 10.1111/cei.13376 PMC6904656

[148] Li D , Wu R , Guo W *et al*. STING‐mediated IFI16 degradation negatively controls type I interferon production. Cell Rep 2019; 29: 1249, e1244–1260. 31665637 10.1016/j.celrep.2019.09.069

[149] Wang PH , Ye ZW , Deng JJ *et al*. Inhibition of AIM2 inflammasome activation by a novel transcript isoform of IFI16. EMBO Rep 2018; 19: e45737. 30104205 10.15252/embr.201845737 PMC6172465

[150] Indramohan M , Stehlik C , Dorfleutner A . COPs and POPs patrol inflammasome activation. J Mol Biol 2018; 430: 153–173. 29024695 10.1016/j.jmb.2017.10.004 PMC5766406

[151] de Almeida L , Khare S , Misharin AV *et al*. The PYRIN domain‐only protein POP1 inhibits inflammasome assembly and ameliorates inflammatory disease. Immunity 2015; 43: 264–276. 26275995 10.1016/j.immuni.2015.07.018 PMC4666005

[152] Ratsimandresy RA , Chu LH , Khare S *et al*. The PYRIN domain‐only protein POP2 inhibits inflammasome priming and activation. Nat Commun 2017; 8: 1–15. 28580931 10.1038/ncomms15556 PMC5465353

[153] Stehlik C , Krajewska M , Welsh K , Krajewski S , Godzik A , Reed JC . The PAAD/PYRIN‐only protein POP1/ASC2 is a modulator of ASC‐mediated nuclear‐factor‐κB and pro‐caspase‐1 regulation. Biochem J 2003; 373: 101–113. 12656673 10.1042/BJ20030304 PMC1223462

[154] Bedoya F , Sandler LL , Harton JA . Pyrin‐only protein 2 modulates NF‐κB and disrupts ASC: CLR interactions. J Immunol 2007; 178: 3837–3845. 17339483 10.4049/jimmunol.178.6.3837

[155] Dorfleutner A , Bryan NB , Talbott SJ *et al*. Cellular pyrin domain‐only protein 2 is a candidate regulator of inflammasome activation. Infect Immun 2007; 75: 1484–1492. 17178784 10.1128/IAI.01315-06 PMC1828547

[156] Lu A , Magupalli VG , Ruan J *et al*. Unified polymerization mechanism for the assembly of ASC‐dependent inflammasomes. Cell 2014; 156: 1193–1206. 24630722 10.1016/j.cell.2014.02.008 PMC4000066

[157] Vajjhala PR , Mirams RE , Hill JM . Multiple binding sites on the pyrin domain of ASC protein allow self‐association and interaction with NLRP3 protein. J Biol Chem 2012; 287: 41732–41743. 23066025 10.1074/jbc.M112.381228 PMC3516722

[158] Periasamy S , Porter KA , Atianand MK *et al*. Pyrin‐only protein 2 limits inflammation but improves protection against bacteria. Nat Commun 2017; 8: 1–12. 28580947 10.1038/ncomms15564 PMC5512670

[159] Khare S , Ratsimandresy RA , De Almeida L *et al*. The PYRIN domain–only protein POP3 inhibits ALR inflammasomes and regulates responses to infection with DNA viruses. Nat Immunol 2014; 15: 343–353. 24531343 10.1038/ni.2829 PMC4123781

[160] Porter KA , Duffy EB , Nyland P , Atianand MK , Sharifi H , Harton JA . The CLRX. 1/NOD24 (NLRP2P) pseudogene codes a functional negative regulator of NF‐κB, pyrin‐only protein 4. Genes Immun 2014; 15: 392–403. 24871464 10.1038/gene.2014.30 PMC4311403

[161] Druilhe A , Srinivasula S , Razmara M , Ahmad M , Alnemri E . Regulation of IL‐1β generation by Pseudo‐ICE and ICEBERG, two dominant negative caspase recruitment domain proteins. Cell Death Differ 2001; 8: 649–657. 11536016 10.1038/sj.cdd.4400881

[162] Lamkanfi M , Denecker G , Kalai M *et al*. INCA, a novel human caspase recruitment domain protein that inhibits interleukin‐1β generation. J Biol Chem 2004; 279: 51729–51738. 15383541 10.1074/jbc.M407891200

[163] Lee SH , Stehlik C , Reed JC . Cop, a caspase recruitment domain‐containing protein and inhibitor of caspase‐1 activation processing. J Biol Chem 2001; 276: 34495–34500. 11432859 10.1074/jbc.M101415200

[164] Karasawa T , Kawashima A , Usui F *et al*. Oligomerized CARD16 promotes caspase‐1 assembly and IL‐1β processing. FEBS Open Bio 2015; 5: 348–356. 10.1016/j.fob.2015.04.011 PMC4420773 25973362

[165] Lu A , Li Y , Schmidt FI *et al*. Molecular basis of caspase‐1 polymerization and its inhibition by a new capping mechanism. Nat Struct Mol Biol 2016; 23: 416–425. 27043298 10.1038/nsmb.3199 PMC4856535

[166] Humke EW , Shriver SK , Starovasnik MA , Fairbrother WJ , Dixit VM . ICEBERG: a novel inhibitor of interleukin‐1β generation. Cell 2000; 103: 99–111. 11051551 10.1016/s0092-8674(00)00108-2

[167] Vigano E , Diamond CE , Spreafico R , Balachander A , Sobota RM , Mortellaro A . Human caspase‐4 and caspase‐5 regulate the one‐step non‐canonical inflammasome activation in monocytes. Nat Commun 2015; 6: 8761. 26508369 10.1038/ncomms9761 PMC4640152

[168] Kayagaki N , Warming S , Lamkanfi M *et al*. Non‐canonical inflammasome activation targets caspase‐11. Nature 2011; 479: 117–U146. 22002608 10.1038/nature10558

[169] Matikainen S , Nyman TA , Cypryk W . Function and Regulation of Noncanonical Caspase‐4/5/11 Inflammasome. J Immunol 2020; 204: 3063–3069. 32513874 10.4049/jimmunol.2000373

[170] Lu M , Zhu K , Zheng Q , Ma X , Zou L . Severe diarrhea in a 10‐year‐old girl with Aicardi‐Goutieres syndrome due to IFIH1 gene mutation. Am J Med Genet A 2021; 185: 3146–3152. 34189822 10.1002/ajmg.a.62397

[171] Goos H , Fogarty CL , Sahu B *et al*. Gain‐of‐function CEBPE mutation causes noncanonical autoinflammatory inflammasomopathy. J Allergy Clin Immunol 2019; 144: 1364–1376. 31201888 10.1016/j.jaci.2019.06.003 PMC11057357

[172] Wiggins KA , Clarke MC . Senescence utilises inflammatory caspases to drive SASP. Aging (Albany NY) 2019; 11: 3891–3892. 31209185 10.18632/aging.102031 PMC6629008

[173] Parry AJ , Narita M . Old cells, new tricks: chromatin structure in senescence. Mamm Genome 2016; 27: 320–331. 27021489 10.1007/s00335-016-9628-9 PMC4935760

[174] Fernandez‐Duran I , Quintanilla A , Tarrats N *et al*. Cytoplasmic innate immune sensing by the caspase‐4 non‐canonical inflammasome promotes cellular senescence. Cell Death Differ 2022; 29: 1267–1282. 34916628 10.1038/s41418-021-00917-6 PMC9177556

[175] McDermott MF , Aksentijevich I , Galon J *et al*. Germline mutations in the extracellular domains of the 55 kDa TNF receptor, TNFR1, define a family of dominantly inherited autoinflammatory syndromes. Cell 1999; 97: 133–144. 10199409 10.1016/s0092-8674(00)80721-7

[176] Lachmann HJ , Papa R , Gerhold K *et al*. The phenotype of TNF receptor‐associated autoinflammatory syndrome (TRAPS) at presentation: a series of 158 cases from the Eurofever/EUROTRAPS international registry. Ann Rheum Dis 2014; 73: 2160–2167. 23965844 10.1136/annrheumdis-2013-204184 PMC4251160

[177] Georgin‐Lavialle S , Fayand A , Rodrigues F , Bachmeyer C , Savey L , Grateau G . Autoinflammatory diseases: State of the art. Presse Med 2019; 48: E25–E48. 30686513 10.1016/j.lpm.2018.12.003

[178] Touitou I , Lesage S , McDermott M *et al*. Infevers: an evolving mutation database for auto‐inflammatory syndromes. Hum Mutat 2004; 24: 194–198. 15300846 10.1002/humu.20080

[179] Nedjai B , Hitman GA , Yousaf N *et al*. Abnormal tumor necrosis factor receptor I cell surface expression and NF‐κB activation in tumor necrosis factor receptor‐associated periodic syndrome. Arthritis Rheum 2008; 58: 273–283. 18163488 10.1002/art.23123

[180] Lobito AA , Kimberley FC , Muppidi JR *et al*. Abnormal disulfide‐linked oligomerization results in ER retention and altered signaling by TNFR1 mutants in TNFR1‐associated periodic fever syndrome (TRAPS). Blood 2006; 108: 1320–1327. 16684962 10.1182/blood-2005-11-006783 PMC1895878

[181] Todd I , Radford PM , Draper‐Morgan KA *et al*. Mutant forms of tumour necrosis factor receptor I that occur in TNF‐receptor‐associated periodic syndrome retain signalling functions but show abnormal behaviour. Immunology 2004; 113: 65–79. 15312137 10.1111/j.1365-2567.2004.01942.x PMC1782552

[182] Bulua AC , Simon A , Maddipati R *et al*. Mitochondrial reactive oxygen species promote production of proinflammatory cytokines and are elevated in TNFR1‐associated periodic syndrome (TRAPS). J Exp Med 2011; 208: 519–533. 21282379 10.1084/jem.20102049 PMC3058571

[183] Dickie LJ , Aziz AM , Savic S *et al*. Involvement of X‐box binding protein 1 and reactive oxygen species pathways in the pathogenesis of tumour necrosis factor receptor‐associated periodic syndrome. Ann Rheum Dis 2012; 71: 2035–2043. 22679299 10.1136/annrheumdis-2011-201197

[184] Yang Q , Kim YS , Lin Y , Lewis J , Neckers L , Liu ZG . Tumour necrosis factor receptor 1 mediates endoplasmic reticulum stress‐induced activation of the MAP kinase JNK. EMBO Rep 2006; 7: 622–627. 16680093 10.1038/sj.embor.7400687 PMC1479600

[185] Bronner DN , Abuaita BH , Chen X *et al*. Endoplasmic reticulum stress activates the inflammasome via NLRP3‐ and caspase‐2‐driven mitochondrial damage. Immunity 2015; 43: 451–462. 26341399 10.1016/j.immuni.2015.08.008 PMC4582788

[186] Lerner AG , Upton JP , Praveen PV *et al*. IRE1α induces thioredoxin‐interacting protein to activate the NLRP3 inflammasome and promote programmed cell death under irremediable ER stress. Cell Metab 2012; 16: 250–264. 22883233 10.1016/j.cmet.2012.07.007 PMC4014071

[187] Jacobelli S , Andre M , Alexandra JF , Dode C , Papo T . Failure of anti‐TNF therapy in TNF receptor 1‐associated periodic syndrome (TRAPS). Rheumatology 2007; 46: 1211–1212. 16935919 10.1093/rheumatology/kel298

[188] De Benedetti F , Gattorno M , Anton J *et al*. Canakinumab for the treatment of autoinflammatory recurrent fever syndromes. N Engl J Med 2018; 378: 1908–1919. 29768139 10.1056/NEJMoa1706314

[189] Coll RC , Robertson AA , Chae JJ *et al*. A small‐molecule inhibitor of the NLRP3 inflammasome for the treatment of inflammatory diseases. Nat Med 2015; 21: 248–255. 25686105 10.1038/nm.3806 PMC4392179

[190] Marchetti C , Swartzwelter B , Gamboni F *et al*. OLT1177, a beta‐sulfonyl nitrile compound, safe in humans, inhibits the NLRP3 inflammasome and reverses the metabolic cost of inflammation. Proc Natl Acad Sci USA 2018; 115: E1530–E1539. 29378952 10.1073/pnas.1716095115 PMC5816172

[191] Kuemmerle‐Deschner JB , Ozen S , Tyrrell PN *et al*. Diagnostic criteria for cryopyrin‐associated periodic syndrome (CAPS). Ann Rheum Dis 2017; 76: 942–947. 27707729 10.1136/annrheumdis-2016-209686

[192] Sarrauste de Menthière C , Terriere S , Pugnere D , Ruiz M , Demaille J , Touitou I . INFEVERS: the Registry for FMF and hereditary inflammatory disorders mutations. Nucleic Acids Res 2003; 31: 282–285. 12520003 10.1093/nar/gkg031 PMC165478

[193] Dowds TA , Masumoto J , Zhu L , Inohara N , Núñez G . Cryopyrin‐induced interleukin 1β secretion in monocytic cells: enhanced activity of disease‐associated mutants and requirement for ASC. J Biol Chem 2004; 279: 21924–21928. 15020601 10.1074/jbc.M401178200

[194] Kubota T , Koike R . Cryopyrin‐associated periodic syndromes: background and therapeutics. Mod Rheumatol 2010; 20: 213–221. 20140476 10.1007/s10165-009-0271-0

[195] Agostini L , Martinon F , Burns K , McDermott MF , Hawkins PN , Tschopp J . NALP3 forms an IL‐1β‐processing inflammasome with increased activity in Muckle‐Wells autoinflammatory disorder. Immunity 2004; 20: 319–325. 15030775 10.1016/s1074-7613(04)00046-9

[196] Stack JH , Beaumont K , Larsen PD *et al*. IL‐converting enzyme/caspase‐1 inhibitor VX‐765 blocks the hypersensitive response to an inflammatory stimulus in monocytes from familial cold autoinflammatory syndrome patients. J Immunol 2005; 175: 2630–2634. 16081838 10.4049/jimmunol.175.4.2630

[197] Brydges SD , Mueller JL , McGeough MD *et al*. Inflammasome‐mediated disease animal models reveal roles for innate but not adaptive immunity. Immunity 2009; 30: 875–887. 19501000 10.1016/j.immuni.2009.05.005 PMC2759865

[198] Tassi S , Carta S , Delfino L *et al*. Altered redox state of monocytes from cryopyrin‐associated periodic syndromes causes accelerated IL‐1β secretion. Proc Natl Acad Sci USA 2010; 107: 9789–9794. 20445104 10.1073/pnas.1000779107 PMC2906851

[199] Rosengren S , Mueller JL , Anderson JP *et al*. Monocytes from familial cold autoinflammatory syndrome patients are activated by mild hypothermia. J Allergy Clin Immunol 2007; 119: 991–996. 17320940 10.1016/j.jaci.2006.12.649 PMC4322003

[200] Brydges SD , Broderick L , McGeough MD , Pena CA , Mueller JL , Hoffman HM . Divergence of IL‐1, IL‐18, and cell death in NLRP3 inflammasomopathies. J Clin Invest 2013; 123: 4695–4705. 24084736 10.1172/JCI71543 PMC3809806

[201] Yu J , Nagasu H , Murakami T *et al*. Inflammasome activation leads to Caspase‐1–dependent mitochondrial damage and block of mitophagy. Proc Natl Acad Sci USA 2014; 111: 15514–15519. 25313054 10.1073/pnas.1414859111 PMC4217429

[202] Xiao J , Wang C , Yao J‐C *et al*. Gasdermin D mediates the pathogenesis of neonatal‐onset multisystem inflammatory disease in mice. PLoS Biol 2018; 16: e3000047. 30388107 10.1371/journal.pbio.3000047 PMC6235378

[203] Abbate A , Toldo S , Marchetti C , Kron J , Van Tassell BW , Dinarello CA . Interleukin‐1 and the inflammasome as therapeutic targets in cardiovascular disease. Circ Res 2020; 126: 1260–1280. 32324502 10.1161/CIRCRESAHA.120.315937 PMC8760628

[204] Coll RC , Hill JR , Day CJ *et al*. MCC950 directly targets the NLRP3 ATP‐hydrolysis motif for inflammasome inhibition. Nat Chem Biol 2019; 15: 556–559. 31086327 10.1038/s41589-019-0277-7

[205] Hoozemans JJ , Veerhuis R , Van Haastert ES *et al*. The unfolded protein response is activated in Alzheimer's disease. Acta Neuropathol 2005; 110: 165–172. 15973543 10.1007/s00401-005-1038-0

[206] Hoozemans JJ , van Haastert ES , Nijholt DA , Rozemuller AJ , Eikelenboom P , Scheper W . The unfolded protein response is activated in pretangle neurons in Alzheimer's disease hippocampus. Am J Pathol 2009; 174: 1241–1251. 19264902 10.2353/ajpath.2009.080814 PMC2671357

[207] Oh J , Huh KY , Cho YG *et al*. Safety, tolerability and pharmacokinetics and pharmacodynamics of HL2351, a novel hybrid fc‐fused interleukin‐1 receptor antagonist, in healthy subjects: A first‐in‐human study. Br J Clin Pharmacol 2020; 86: 372–379. 31658396 10.1111/bcp.14161 PMC7015738

[208] Gordon D , Hellriegel ET , Hope HR , Burt D , Monahan JB . Safety, tolerability, pharmacokinetics, and pharmacodynamics of the MK2 inhibitor ATI‐450 in healthy subjects: a placebo‐controlled, randomized phase 1 study. Clin Pharmacol 2021; 13: 123–134. 34140814 10.2147/CPAA.S305308 PMC8203602

[209] Reimann HA . Periodic disease; a probable syndrome including periodic fever, benign paroxysmal peritonitis, cyclic neutropenia and intermittent arthralgia. JAMA 1948; 136: 239–244. 10.1001/jama.1948.02890210023004 18920089

[210] Siegal S . Benign paroxysmal peritonitis. Gastroenterology 1949; 12: 234–247. 18124924

[211] French FMFC . A candidate gene for familial Mediterranean fever. Nat Genet 1997; 17: 25–31. 9288094 10.1038/ng0997-25

[212] Aksentijevich I , Centola M , Deng ZM *et al*. Ancient missense mutations in a new member of the RoRet gene family are likely to cause familial Mediterranean fever. Cell 1997; 90: 797–807. 9288758 10.1016/s0092-8674(00)80539-5

[213] Ozen S , Batu ED , Demir S . Familial mediterranean fever: recent developments in pathogenesis and new recommendations for management. Front Immunol 2017; 8: 253. 28386255 10.3389/fimmu.2017.00253 PMC5362626

[214] Papin S , Cuenin S , Agostini L *et al*. The SPRY domain of Pyrin, mutated in familial Mediterranean fever patients, interacts with inflammasome components and inhibits proIL‐1beta processing. Cell Death Differ 2007; 14: 1457–1466. 17431422 10.1038/sj.cdd.4402142

[215] Ozen S , Demirkaya E , Erer B *et al*. EULAR recommendations for the management of familial Mediterranean fever. Ann Rheum Dis 2016; 75: 644–651. 26802180 10.1136/annrheumdis-2015-208690

[216] Van Gorp H , Saavedra PH , de Vasconcelos NM *et al*. Familial Mediterranean fever mutations lift the obligatory requirement for microtubules in Pyrin inflammasome activation. Proc Natl Acad Sci USA 2016; 113: 14384–14389. 27911804 10.1073/pnas.1613156113 PMC5167202

[217] Moghaddas F , Llamas R , De Nardo D *et al*. A novel pyrin‐associated autoinflammation with neutrophilic dermatosis mutation further defines 14‐3‐3 binding of pyrin and distinction to familial mediterranean fever. Ann Rheum Dis 2017; 76: 2085–2094. 28835462 10.1136/annrheumdis-2017-211473 PMC5687562

[218] Masters SL , Lagou V , Jeru I *et al*. Familial autoinflammation with neutrophilic dermatosis reveals a regulatory mechanism of pyrin activation. Sci Transl Med 2016; 8: 332ra345. 10.1126/scitranslmed.aaf1471 27030597

[219] Liu X , Xia S , Zhang Z , Wu H , Lieberman J . Channelling inflammation: gasdermins in physiology and disease. Nat Rev Drug Discov 2021; 20: 384–405. 33692549 10.1038/s41573-021-00154-z PMC7944254

[220] Kanneganti A , Malireddi RKS , Saavedra PHV *et al*. GSDMD is critical for autoinflammatory pathology in a mouse model of Familial Mediterranean Fever. J Exp Med 2018; 215: 1519–1529. 29793924 10.1084/jem.20172060 PMC5987922

[221] Xiao J , Wang C , Yao JC *et al*. Gasdermin D mediates the pathogenesis of neonatal‐onset multisystem inflammatory disease in mice. PLoS Biol 2018; 16: e3000047. 30388107 10.1371/journal.pbio.3000047 PMC6235378

[222] Dinarello CA . Overview of the IL‐1 family in innate inflammation and acquired immunity. Immunol Rev 2018; 281: 8–27. 29247995 10.1111/imr.12621 PMC5756628

